# Coupling of *Rigor Mortis* and Intestinal Necrosis during *C. elegans* Organismal Death

**DOI:** 10.1016/j.celrep.2018.02.050

**Published:** 2018-03-06

**Authors:** Evgeniy R. Galimov, Rosina E. Pryor, Sarah E. Poole, Alexandre Benedetto, Zachary Pincus, David Gems

**Affiliations:** 1 Institute of Healthy Ageing and Department of Genetics, Evolution and Environment, University College London, London, UK; 2 Division of Biomedical and Life Sciences, Faculty of Health and Medicine, Lancaster University, Lancaster LA1 4YW, UK; 3 Department of Genetics and Department of Developmental Biology, Washington University in St. Louis, St. Louis, MO 63110, USA

**Keywords:** aging, ATP, calcium, *C. elegans*, muscle, necrosis, organismal death, pathology, *rigor mortis*

## Abstract

Organismal death is a process of systemic collapse whose mechanisms are less well understood than those of cell death. We previously reported that death in *C. elegans* is accompanied by a calcium-propagated wave of intestinal necrosis, marked by a wave of blue autofluorescence (death fluorescence). Here, we describe another feature of organismal death, a wave of body wall muscle contraction, or death contraction (DC). This phenomenon is accompanied by a wave of intramuscular Ca^2+^ release and, subsequently, of intestinal necrosis. Correlation of directions of the DC and intestinal necrosis waves implies coupling of these death processes. Long-lived insulin/IGF-1-signaling mutants show reduced DC and delayed intestinal necrosis, suggesting possible resistance to organismal death. DC resembles mammalian *rigor mortis*, a postmortem necrosis-related process in which Ca^2+^ influx promotes muscle hyper-contraction. In contrast to mammals, DC is an early rather than a late event in *C. elegans* organismal death.

**Video Abstract:**

## Highlights

• A wave of body wall muscle contraction occurs during *C. elegans* organismal death
• This *rigor mortis*-like phenomenon is coupled to a wave of intestinal necrosis
• Both waves are accompanied by Ca^2+^ release and a drop in ATP levels
• Properties of long-lived *daf-2* mutants suggest resistance to organismal death

## Graphical Abstract

**Figure:**
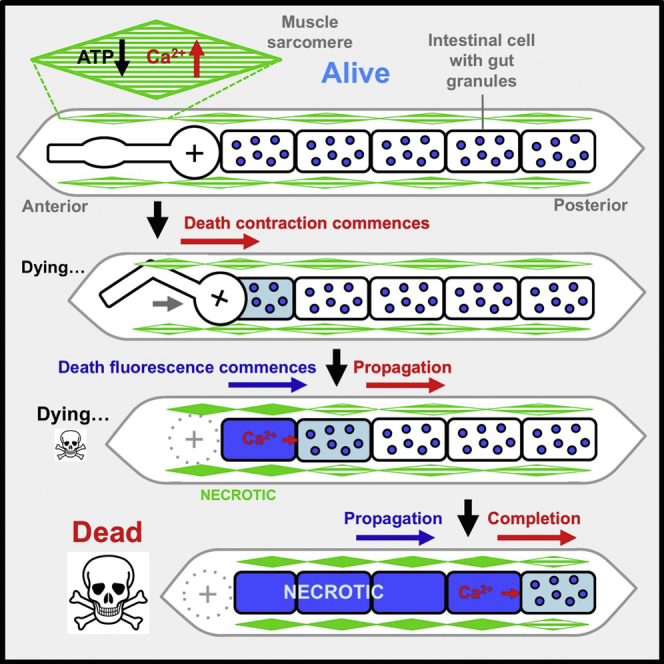

## Teaser

Galimov et al. describe mechanisms of organismal death in *C. elegans*. They document a *rigor mortis*-like wave of muscle hyper-contraction accompanied by Ca^2+^ release and falling ATP, which is coupled to the previously described wave of intestinal necrosis in a process that resembles a distorted and deadly defecation cycle.

## Introduction

What is death? Viewed from a medical or legal perspective, death is the “permanent cessation of the critical functions of the organism as a whole” (Bernat, 1998), and the criteria for defining human death are circulatory system failure and brain death (Bernat, 2013). However, from a biological perspective, death appears quite different, in that the body of the person just declared dead actually remains full of life. At that point, most of the cells and organs are still alive (as demonstrated by the efficacy of organ transplants from cadavers), and the timing of the death of specific organs and tissues after legal death varies considerably, due to differing vulnerability to hypoxia (Knight et al., 1997). Moreover, complex postmortem changes in gene expression have been recorded (Pozhitkov et al., 2017). Thus, in biological terms, death is more a process than an event (Morison, 1971).

The mechanisms of organismal death, how it happens and how it is triggered, its exact beginning and ending, are a neglected topic within biology yet important for understanding fatal diseases, including those caused by aging. Although many major causes of death involve visible pathology (e.g., cancers), how exactly many types of pathology lead to death is unclear (with the notable exception of cardiovascular and cerebrovascular pathologies). Death can also occur without easily identifiable causes, particularly in the elderly, where it is often ascribed to “death of old age” (Meadows, 2007). Likewise, the exact causes of death in senescent laboratory rodents are often unclear, with postmortem (necropsy) studies failing to detect pathology in up to 10%–30% of mice (Maronpot, 1999, Son, 2003). Furthermore, in many cases the presence of pathology can prove only that the animal died with the pathology, not because of it. Studying the biology of organismal death is worthwhile since the knowledge obtained can help us to understand how both senescent and non-senescent pathologies cause organismal death and to devise treatments to bring back critically ill patients from the brink of death.

The nematode *Caenorhabditis elegans* is a convenient model organism for the study of complex biological processes, and it is well suited for investigations of organismal death. This is particularly relevant to the ongoing endeavor of understanding the biology of aging using this organism. While numerous long-lived *C. elegans* mutants have been isolated and molecular pathways influencing lifespan discovered (Kenyon, 2010, Lapierre and Hansen, 2012), the causes of the increase in mortality rate during aging in *C. elegans* remain unclear. As the terminal event that determines lifespan, it is important to understand organismal death and how it is triggered by senescent pathology.

Our previous study described the occurrence during *C. elegans* organismal death of a calcium-propagated wave of necrotic cell death in the intestine, typically in an anterior-to-posterior (AP) direction (Coburn et al., 2013). Under UV light, this wave is rendered visible as a wave of blue autofluorescence (death fluorescence [DF]), caused by the release of tryptophan-derived anthranilates from degenerating lysosome-related organelles (Coburn et al., 2013, Zhang et al., 2016a). It was once thought that the age increase in intestinal autofluorescence reflects the accumulation of the damage product lipofuscin, but several observations argue against this interpretation (Coburn et al., 2013, Coburn and Gems, 2013, Pincus et al., 2016).

Organismal death in *C. elegans* is also accompanied by changes in body volume, with an initial reduction in size followed by recovery of pre-death body size (Stroustrup et al., 2013). An interesting possibility is that this phenomenon is related to *rigor mortis*, another Ca^2+^ release-driven death-related process. In *rigor mortis* (stiffness of death), there occurs a transient postmortem muscle contraction that results from the biochemical changes that take place in dying muscle cells. In forensic science, assessment of *rigor mortis* can help to estimate time of death (Mathur and Agrawal, 2011). *Rigor mortis* is also of interest to the meat industry since its onset and resolution underlies the process of meat tenderization (Huff Lonergan et al., 2010, Paredi et al., 2012). *Rigor mortis* has been studied previously in several mammalian species, but not in invertebrates.

The immediate cause of muscle contraction during *rigor mortis* appears to be ATP depletion (Bate-Smith and Bendall, 1947, Kawai and Brandt, 1976). In normal muscle physiology, Ca^2+^ ions are released from the sarcoplasmic reticulum (SR) to initiate the muscle contraction cycle. During relaxation, calcium is pumped back into the SR via ATP-dependent channels (Slack et al., 1997). After death, when respiration in muscles becomes impossible due to the lack of oxygen circulation, the principal sources of ATP become glycolysis and creatine phosphate stores (Bate-Smith and Bendall, 1956). When these are depleted, Ca^2+^ cannot be pumped back due to a lack of ATP and muscles become chronically contracted (Jeacocke, 1993). Postmortem relaxation of muscle is also promoted by increased Ca^2+^ levels, which induce degradation of muscle cell constituents by Ca^2+^ proteases (calpains) (Koohmaraie, 1992). It is notable that both *rigor mortis* and necrosis are promoted by increased Ca^2+^ levels that lead ultimately to proteolytic destruction of the cell.

Here we describe the occurrence of a *rigor mortis*-like process in *C. elegans*. We report that a transient reduction in body length occurs during death in young adult worms killed using lethal stress and during death from old age. We show that such death contraction is altered by manipulations of muscle function in a manner consistent with the occurrence of *rigor mortis* during *C. elegans* organismal death. Death contraction also precedes, and is coupled with, intestinal necrosis. This suggests that an organism-wide wave of Ca^2+^ release causes *rigor mortis* and intestinal necrosis and is a major event driving organismal death in *C. elegans*. We also find that death contraction is suppressed in long-lived *daf-2* insulin/IGF-1 receptor mutants, suggesting possible organismal death resistance, and that this suppression requires *daf-18*/PTEN, but not *daf-16*/FoxO.

## Results

### Death in *C. elegans* Is Accompanied by an AP Wave of Contraction

Animals dying from old age typically exhibit DF, marking intestinal necrosis (Coburn et al., 2013), and also a transient reduction in body size (Stroustrup et al., 2013). Since such necrosis and DF also occur during stress-induced death in young adults, we asked whether they also show the death-induced reduction in body size. Changes in worm body length were captured and converted into kymographs (single images displaying temporal changes) (Nair et al., 2014, Zhou et al., 2001), from which data were extracted with MATLAB and analyzed using R scripts (Experimental Procedures).

**Figure 1:**
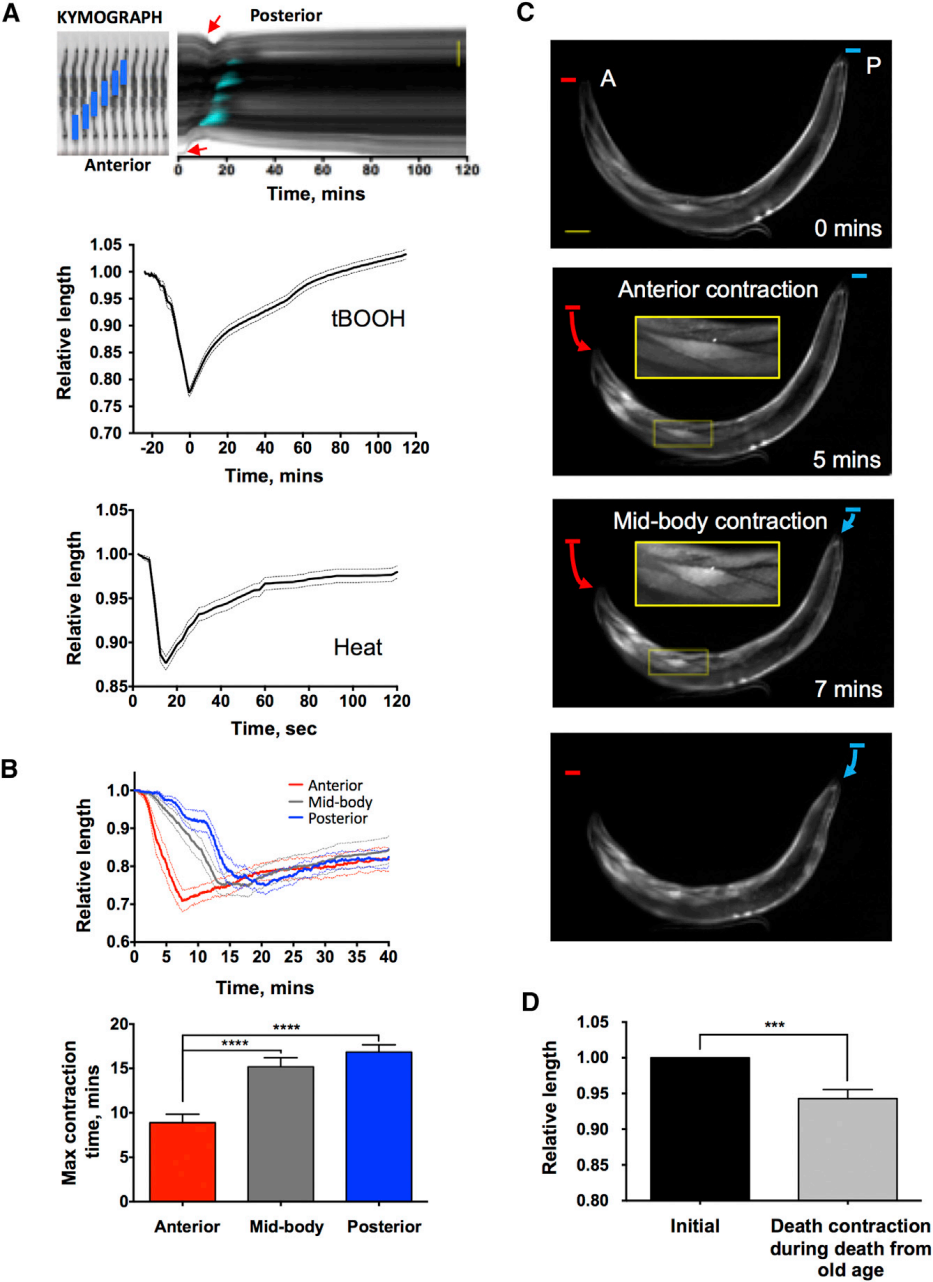
DC Occurs in an AP Wave (A) Body contraction during death is accompanied by DF. Top: kymograph is shown (tBOOH-induced death); top left panel clarifies what the kymograph represents (blue bars, death fluorescence moving along the worm; red arrows, onset of contraction). Scale bar, 200 μm. Bottom: plots show mean body length against time after death induced by tBOOH (n = 29; pooled from 5 experiments) or heat stress (n = 18; pooled from 5 experiments). For tBOOH killing, the point of maximal contraction was set as time point 0. (B) Body contraction during tBOOH killing occurs in an AP wave. Top: plot shows mean length ± SEM in anterior, mid-body, and posterior regions against time. Bottom: time of maximal contraction of different parts of nematode body is shown (n = 15, pooled from 5 experiments; one-way ANOVA). (C) Wave of muscle contraction during tBOOH-induced death, muscle fibers visualized using *pmyo-3::GFP*. Scale bar, 50 μm. (D) DC in worms dying of aging, expressed as relative length decrease (n = 19, pooled from 16 experiments; paired t test). Mean ± SEM. ^∗∗∗^p < 0.001 and ^∗∗∗∗^p < 0.0001. See also Figures S1, S2, and S6.

Upon killing with an organic peroxide, *tert*-butylhydroperoxide (tBOOH, 14%), young wild-type (N2) adults exhibited a transient reduction in body length shortly after cessation of movement (Figure 1A) that progressed in an AP wave (Figure 1B; Figure S1). This reduction in body length was accompanied by a wave of DF (Figure 1A; Movie S1). It was previously shown that nose muscle contraction occurs in response to a number of drugs, including fluoxetine (Prozac, a selective serotonin reuptake inhibitor) (Choy and Thomas, 1999). However, this phenomenon is distinct from the effect of tBOOH-induced killing: fluoxetine-induced nose contraction was not accompanied by DF, and worms were still alive 1 hr after treatment (Figure S2). We next killed worms with thermal injury, induced using a thermoelectrically heated microscope stage (PE120, Linkam Scientific). Again, a transient reduction in body length was seen, which occurred at a faster rate than in response to tBOOH (Figure 1A). However, here AP progression was not detected in either the contraction or the DF (data not shown).

We then examined worms dying as the result of aging. Here again, a reduction in body length was seen, consistent with previous observations (Stroustrup et al., 2013). However, the magnitude of the contraction was smaller than in young adults, and it was not seen in all animals (Figure 1D; Movie S2). These results imply that the occurrence of transient body length reduction at death is a typical feature of organismal death in *C. elegans*. For convenience, we will refer to the death-associated reduction in body length as death contraction (DC).

### DC Is Caused by Muscle Contraction

**Figure 2:**
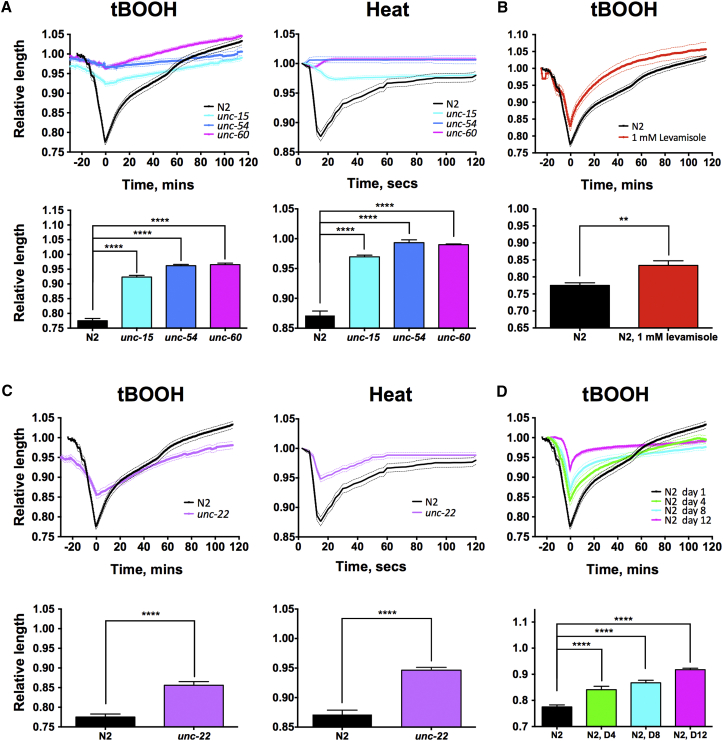
DC Is Caused by Muscle Contraction (A) Suppression of DC by *unc-15(e73)*, *unc-54(e190)*, and *unc-60(e723)*. Death from tBOOH (left) and heat stress (right) is shown. (B) Reduced DC in worms pre-treated with levamisole. (C) Reduced DC in *unc-22(e66)* mutants. Death from tBOOH (left) and heat stress (right) is shown. (D) Effects of age on DC during tBOOH-induced death. Relative length: length relative to that before death. (A–D) Top, DC curves; bottom, maximal DC. n = 13–41, pooled from 3–8 experiments. Mean ± SEM. ^∗∗^p < 0.01 and ^∗∗∗∗^p < 0.0001, one-way ANOVA.

Labeling of body wall muscles with fluorescent myosin, MYO-3::GFP, revealed a clear AP wave of muscle contraction during death (Figure 1C; Movie S1), implying that DC is caused by muscle contraction. To verify this, we tested the effect of altering muscle function on DC and recovery. We first tested whether DC was suppressed in mutants incapable of muscle contraction due to a loss of structural components of the musculature, namely *unc-15(e73)* (paramyosin), *unc-54(e190)* (myosin heavy chain), and *unc-60(e723)* (cofilin). All largely suppressed DC after killing with tBOOH or with heat (Figure 2A).

Then we tested the prediction that DC should be reduced in worms in which muscles are already hyper-contracted by using levamisole, an acetylcholine agonist that binds to receptors in the body wall muscle, causing hyper-contraction. Pre-treatment with 1 mM levamisole shortened body length, and pre-treated worms showed reduced DC (Figure 2B). DC was also reduced in *unc-22(e66)* (twitchin) mutants, which, due to abnormal muscle structure, are already hyper-contracted (Benian et al., 1989) (Figure 2C).

Another characteristic of *rigor mortis* in mammals is that it becomes less marked with age, due to an age-related decline in muscle function (Siegel et al., 2000). Given that senescent decline of muscle (sarcopenia) also occurs in *C. elegans* (Herndon et al., 2002), we tested the effect of aging on DC by examining the effects of tBOOH-induced death in adult worms at different ages. This revealed a marked and progressive decline with increasing age in the magnitude of DC (Figure 2D). Taken together, these findings clearly imply that *C. elegans* DC is, like mammalian *rigor mortis*, the result of muscle contraction occurring during death.

### Correlation between DC and DF Wave Patterns

The occurrence of DC could provide insight into other aspects of *C. elegans* organismal death. Dying worms typically exhibit an AP wave of intestinal fluorescence, marking an AP wave of intestinal necrosis (Coburn et al., 2013, Zhang et al., 2016a). It has hitherto been unclear how this wave is initiated or how its AP polarity specified. To investigate a possible link between the AP waves of muscle contraction and intestinal necrosis, we examined the correspondence between DC and DF waves during tBOOH-induced death at different ages and during death from old age.

Analysis of death in older worms revealed the occurrence of additional forms of DC and DF wave. In addition to anterior-to-posterior DC waves (DC AP), we also observed posterior-to-anterior (DC PA) and simultaneous anterior and posterior contraction (DC S). Similarly, there were a variety of DF wave types in addition to the anterior-to-posterior DF waves (DF AP) seen in young adults: in old worms we also saw posterior-to-anterior waves (DF PA), waves originating in the mid-body and propagating outward (DF M), and complex waves with simultaneous AP and PA components (DF AP + PA), or where DF AP (or DF PA) and DF M occurred within the same worm (DF AP [or PA] + M).

**Figure 3:**
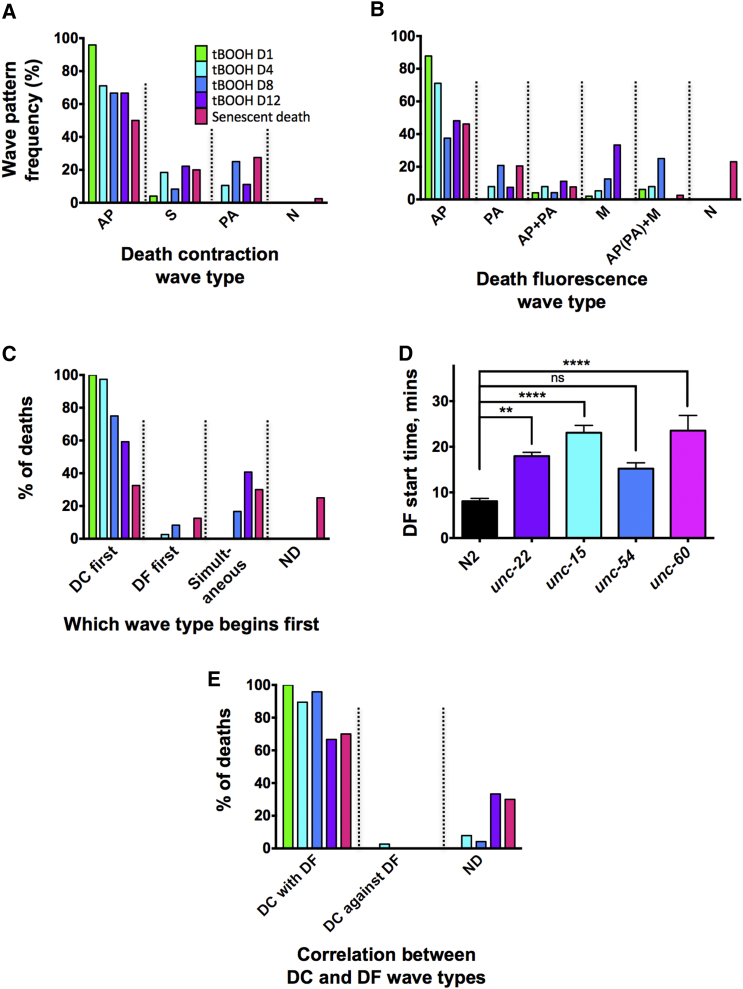
DC Waves Correlate with but Precede DF (A and B) Anterior-to-posterior (AP) waves, posterior-to-anterior (PA) waves, waves starting from the middle (M), or combined wave types in N2 worms killed with tBOOH at different ages, or dying of old age. (A) DC is shown. (B) DF is shown. (C) Which death wave occurs first? DC before DF, DF before DC, simultaneous DC and DF, and undetermined (in the case of M DF) are shown. (D) Blocking DC delays DF (^∗∗^p < 0.01 and ^∗∗∗∗^p < 0.0001, one-way ANOVA). This suggests that DC promotes DF. An alternative possibility that cannot be excluded is that the delay in DF is due to reduced tBOOH ingestion rate, resulting from the slightly reduced pharyngeal pumping rate in these mutants (Figure S3C). (E) Correlation between direction of DC and DF (n = 13–41, pooled from 3–8 experiments). For (A) and (B), N is no contraction or no increase in fluorescence. For (C) and (E), ND is not determined. See also Figures S3 and S4.

While 96% of young adults exhibited DC AP waves during tBOOH-induced killing, this proportion decreased progressively with age, while in those dying from senescence, DC AP waves were detectable in only 50% of cases (Figure 3A). Meanwhile, DC S and DC PA waves occurred more frequently in older tBOOH-killed animals and during death from senescence (Figure 3A). Similarly, 89% of young adults killed with tBOOH showed DF AP waves, decreasing to 46% in worms dying from old age, while the frequency of DF PA, DF M, and complex waves increased with age (Figure 3B; Figure S3A). These results imply that aging alters the process of organismal death.

We also examined DC and DF in senescent worms cultured in a custom automated vermiculture system (Zhang et al., 2016b). For this we used the strain AQ2953 (described below) grown at 25°C and rendered sterile by *pos-1* RNAi. Under these conditions, DC was less pronounced, but contraction and DF at the anterior or posterior end or both were detected (Figures S4A–S4C).

In killed young adults, DC typically started immediately prior to DF, while in older killed animals (days 8 and 12) or in those dying from senescence, DC and DF often occurred simultaneously (Figure 3C). Moreover, in killed contraction-defective *unc-15*, *unc-54*, and *unc-60* mutants, the appearance of DF was delayed, relative to both the time of exposure to tBOOH (Figure 3D; Table S1) and the time of onset of DC (Figure S4D). This not only suggests that DC triggers DF but also that DF can occur in the absence of DC. Interestingly, the direction of DC and DF waves was strongly correlated, during both tBOOH-induced death and senescence-induced death, both on plates and in the automated system (Figure 3E; Figures S3B and S4C), implying that DC and DF waves are coupled.

Overall, these results suggest that organismal death can involve a two-step process (particularly in younger adults), in which DC occurs first and then triggers DF. Here either the orientation of the DC wave determines that of the DF wave or there exists an underlying determinant of the orientation of both waves. Supporting the latter interpretation, AP orientation of DF was still present when DC was reduced using *unc* mutations (Figure S4E).

### A Wave of Ca^2+^ Release in Body Wall Muscle during DC

The earlier onset of DC and the close correspondence between the direction of DC and DF suggest that death could somehow be transmitted from the body wall muscles to the intestine. Our findings suggest two possible mechanisms by which intestinal necrosis might be triggered: mechanical stress as the pharynx is driven backward into the anterior intestine or effects of elevated Ca^2+^ levels in the cytoplasm of muscle cells adjacent to the anterior intestine.

**Figure 4:**
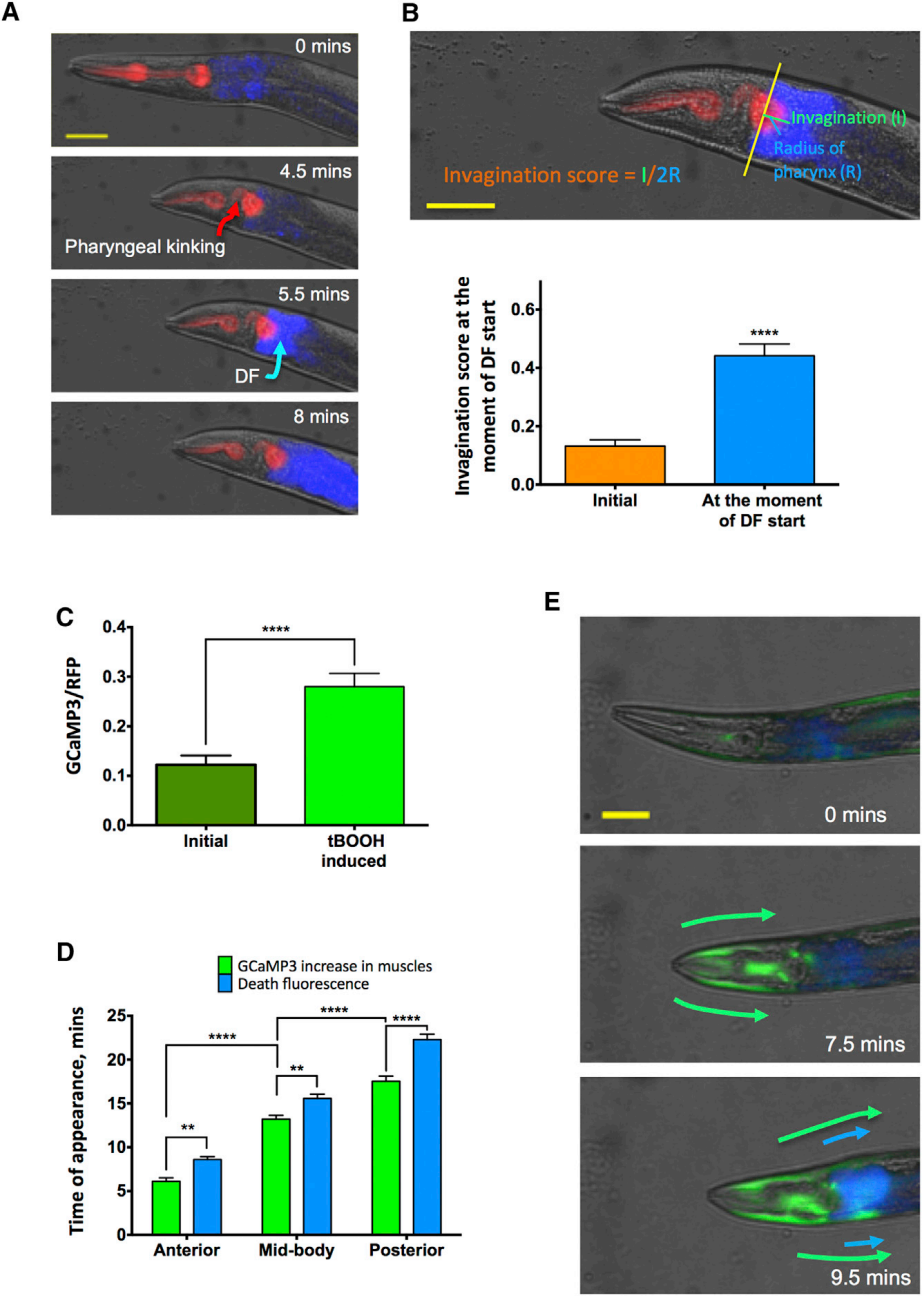
Possible Mechanisms of Propagation of Organismal Death (A) Representative image series of DC driving the pharynx into the anterior intestine with impact coinciding with the initiation of DF. Scale bar, 50 μm. (B) Estimate of the extent of pharyngeal movement into the anterior intestine before DF is initiated. Invagination score (IS = I/2R, where I is length of invagination and R is pharyngeal radius) scheme and estimate (n = 24, pooled from 15 experiments; paired t test) are shown. (C) Increased GCaMP3 fluorescence in anterior body wall muscle cytoplasm during tBOOH-induced death (n = 10, pooled from 2 experiments; paired t test). (D) Time of appearance of Ca^2+^ increase in body wall muscles and DF in intestine in anterior, middle, and posterior parts of worm body during tBOOH-induced death (n = 15, pooled from 2 experiments; two-way ANOVA with Tukey’s HSD correction). (E) Image series of worms showing AP GCaMP3 (Ca^2+^) and DF waves (progression indicated by arrows). Note the former precedes the latter. Scale bar, 50 μm. Mean ± SEM. ^∗∗^p < 0.01 and ^∗∗∗∗^p < 0.0001. See also Figures S4 and S6.

The first possibility is that DC exerts mechanical stress upon the anterior intestine. We noticed that, during organismal death, hyper-contraction of longitudinal body wall muscles in the head pushes the pharynx backward into the anterior intestine. Observation of dying young adult worms (n = 24) showed that, in all cases, shortening of the head caused the pharynx to kink (Figure 4A). Notably, the impact of pharynx with the anterior intestine coincided with the first appearance of DF near the point of impact (Figure 4A; Movie S3). Prior to death, 13% of the posterior bulb diameter was within the anterior intestine, but, at the point of initiation of DF, this rose to 44% (mean value) (Figure 4B). This suggests that mechanical stress could trigger the wave of intestinal necrosis in a belly punch-type effect. An analysis of the relationship between extent of pharyngeal invagination and the timing of first appearance of DF supports this idea (Figure 4B). Pharyngeal invagination could act alone or in combination with pressure on the gut caused by body wall muscle contraction.

Another possibility is that coupling of the DC and DF waves is mediated by a Ca^2+^ signal. In mammals, *rigor mortis* is triggered by a sudden increase in intracellular Ca^2+^ concentration as ATP-dependent Ca^2+^ pumps in the SR are no longer maintained (Jeacocke, 1993). To explore whether a similar mechanism might cause DC in *C. elegans*, we monitored Ca^2+^ release from the SR of body wall muscle at death using the strain AQ2953, which expresses the fluorescent Ca^2+^ sensor GCaMP3 (Butler et al., 2015, Schwarz et al., 2012). Induction of death in young adults (tBOOH) caused a large increase in cytoplasmic Ca^2+^ in body wall muscle (Figure 4C), which occurred in an AP wave that coincided with DC (Figures 4D and 4E; Movie S4). Furthermore, in all worms tested (n = 15), the AP intramuscular Ca^2+^ wave preceded the DF wave (Figures 4D and 4E; Movie S4).

We also examined body wall muscle Ca^2+^ in animals dying of old age in an automated vermiculture system. Here again, Ca^2+^ release typically occurred simultaneously with DC but preceded DF (Figure S4F). In this case, the frequency of AP and PA Ca^2+^ waves was similar (Figure S4G) and both usually co-located with DC, though in some cases Ca^2+^ waves occurred without detectable DC (Figures S4F and S4H).

Thus, during *C. elegans* organismal death, as in mammalian *rigor mortis*, DC of muscle is accompanied by Ca^2+^ release into muscle cytoplasm. This intramuscular Ca^2+^ wave precedes the DF wave, which itself is accompanied by a wave of Ca^2+^ influx in the intestine (Coburn et al., 2013). Our results imply that the two Ca^2+^ waves are coupled, and they suggest that the Ca^2+^ muscle wave might trigger the intestinal Ca^2+^ wave.

### A Wave of ATP Depletion during DC

**Figure 5:**
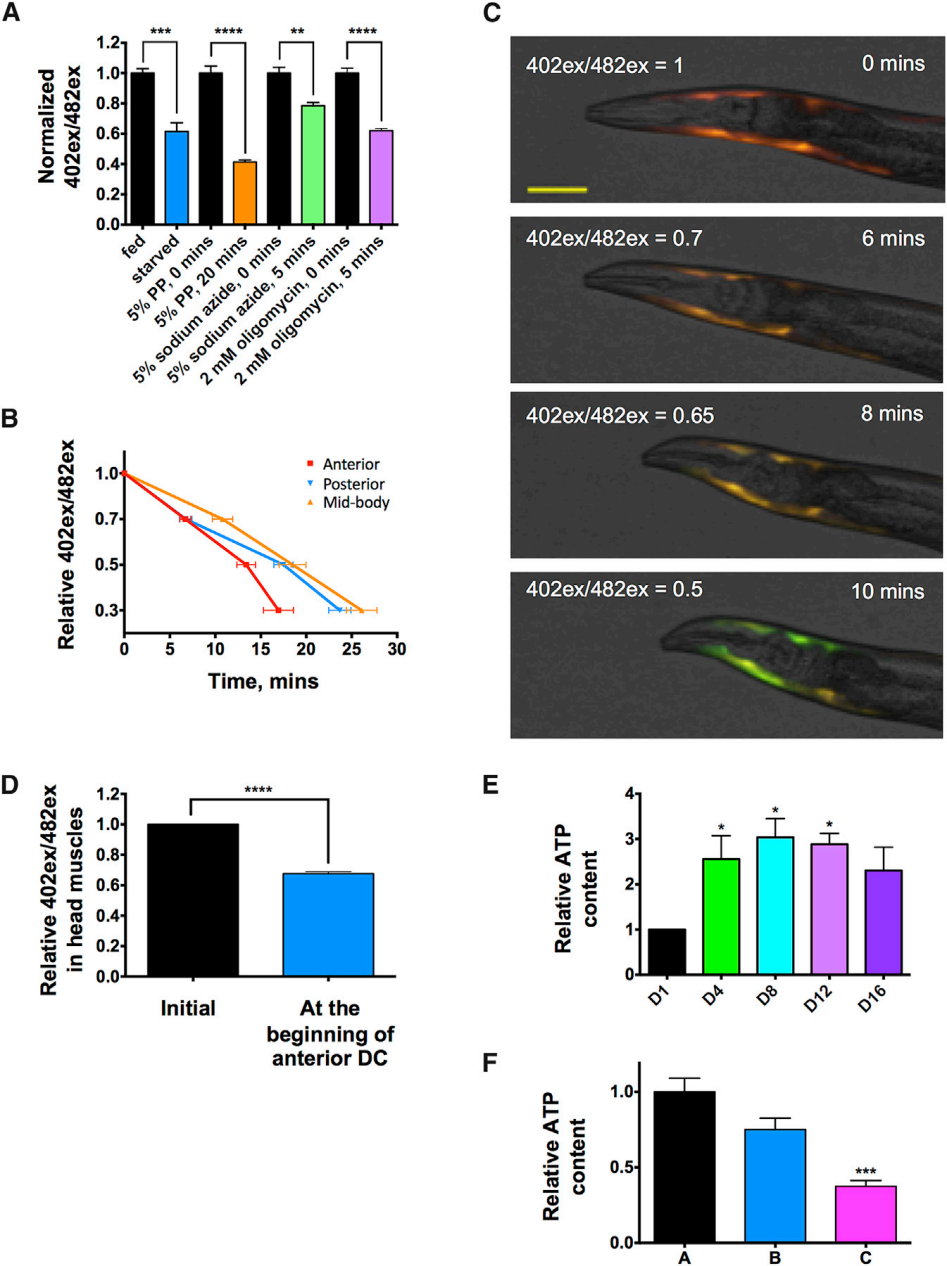
ATP Levels Drop during Organismal Death (A) Relative ATP content in muscle measured with muscle-expressed ATP sensor Queen-2m as 402/482 excitation ratio. Queen-2m signal decreases upon starvation or exposure to 5% 1-phenoxy-2-propanol, 5% sodium azide, or 2 mM oligomycin A (positive control for reporter function) (n = 10–17, pooled from 2–3 experiments; multiple t tests with Bonferroni correction). (B) ATP decline in muscles in different segments of the nematode body upon tBOOH killing, measured with Queen-2m (n = 10, pooled from 2 experiments; two-way ANOVA with Tukey’s HSD correction). (C) Image sequence showing drop in 402/482 excitation ratio (ATP level) during organismal death. Scale bar, 50 μm. (D) 402/482 excitation ratio decline in anterior muscles measured upon tBOOH killing at the beginning of anterior DC (n = 10, pooled from 2 experiments; paired t test). (E) ATP content in class A worms of different ages (1 measurement for 5 worms per time point, 3 trials; one-way ANOVA). (F) ATP content in class A, B, and C worms (n = 10–30, 2 trials; one-way ANOVA). Values represent mean ± SEM. ^∗^p < 0.05, ^∗∗^p < 0.01, ^∗∗∗^p < 0.001, and ^∗∗∗∗^p < 0.0001. See also Figure S5.

Mammalian *rigor mortis* is triggered by ATP depletion (Bate-Smith and Bendall, 1947, Kawai and Brandt, 1976). To test whether DC is accompanied by ATP depletion, we constructed a transgenic *C. elegans* strain with a fluorescent reporter of ATP levels, Queen-2m (Yaginuma et al., 2014), expressed in body wall muscle using the *myo-3* promoter. This sensor, not previously used in *C. elegans*, is composed of circularly permuted EGFP (cpEGFP) inserted between 2 α helices of the bacterial F_o_F_1_-ATP synthase ε subunit, and it acts as a ratiometric probe where the ATP level is proportional to the 402/482 excitation ratio. This strain showed a reduced 402/482 excitation ratio upon starvation or treatment with phenoxy-2-propanol, sodium azide, or oligomycin, in each case consistent with the expected reduction in ATP levels (Figure 5A), thus validating Queen-2m as an ATP sensor in *C. elegans.*

In young adults, tBOOH-induced killing resulted in a wave of ATP depletion, which included a major AP component (Figures 5B and 5C; Movie S5). Moreover, the onset of DC in the head coincided with a 402/482 excitation decrease of 32% (mean) (Figure 5D) and preceded DF (p < 0.0001, paired t test). The simultaneous occurrence of DC and declining ATP is consistent with *rigor mortis*. However, it remains unclear whether the rapid decline in ATP in muscle during organismal death causes DC.

*C. elegans* in liquid culture shows an age-related decline in ATP content (Braeckman et al., 1999), suggesting that a senescent decline in ATP might eventually trigger DC and organismal death. To test this, we first measured ATP content per worm at different ages in plate-cultured animals using a biochemical assay. Here, in contrast to liquid-cultured animals, ATP content remained relatively constant for much of adult life (Figure 5E). Thus, *C. elegans* is capable of maintaining ATP levels up to an advanced age.

Aging *C. elegans* may be classified on the basis of level of motility, where class A animals move normally, class B animals show reduced movement but are capable of crawling if prodded, and class C animals barely move (Herndon et al., 2002). Class C worms are near death and usually die within 1–3 days. Comparison of ATP content in class A, B, and C worms showed a modest decline in class B but a major decline in class C (Figure 5F), likely reflecting the presence of severe, terminal pathology and incipient organismal death. Thus, in plate-cultured *C. elegans*, declining ATP level is apparently more a function of terminal pathology and organismal death than of senescence more broadly.

### Insulin/IGF-1 Signaling Promotes DC

**Figure 6:**
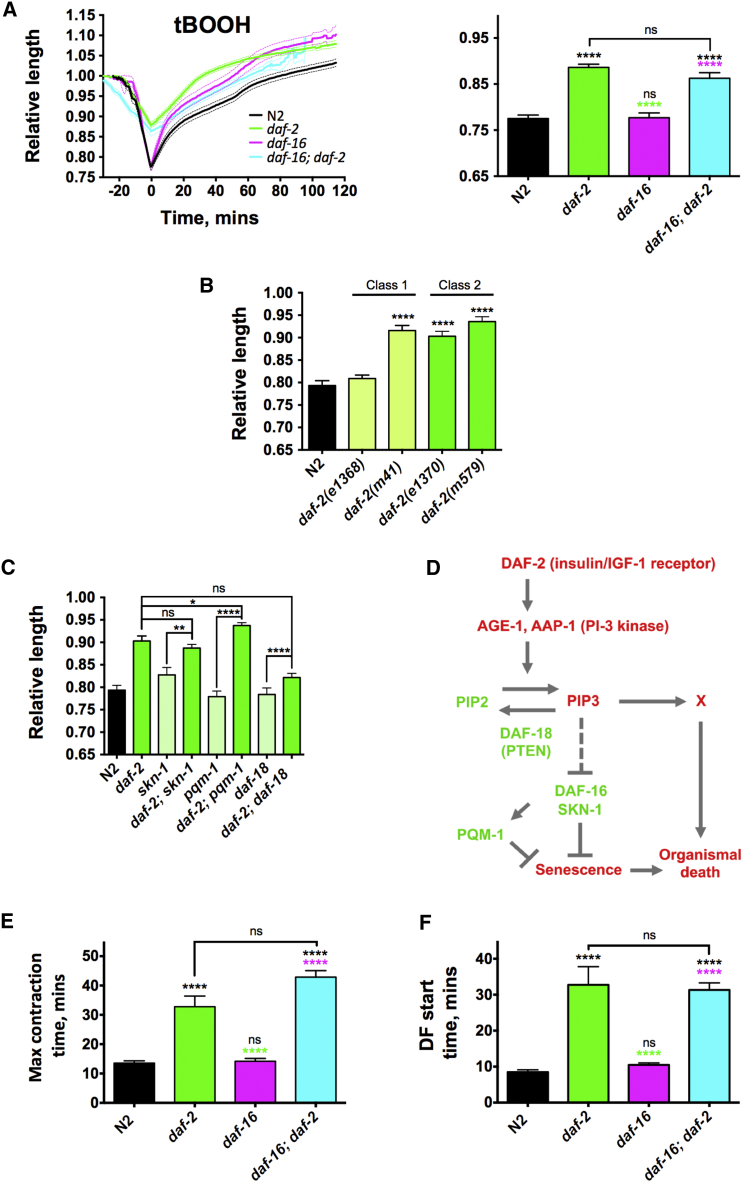
*daf-2* Mutants Are Resistant to DC (A) Effects of *daf-2(e1370)*, *daf-16(mgDf50)*, and *daf-16; daf-2* on DC during death by tBOOH. Left, DC curves; right, maximal DC. (B) Effects of class 1 versus class 2 *daf-2* alleles on DC. (C) Effects of other *daf-2* effectors on *daf-2* DC resistance (all *daf-2* strains are significantly different to N2, p < 0.0001). (D) Scheme: influence of *daf-2* on senescence and organismal death. (E and F) Effects of *daf-2*, *daf-16*, and *daf-16; daf-2* on timing of DC (E) and DF (F) after lethal tBOOH exposure. n = 17–29, pooled from 3–8 experiments. Mean ± SEM. ^∗^p < 0.05, ^∗∗^p < 0.01, and ^∗∗∗∗^p < 0.0001, one-way ANOVA with Tukey’s HSD correction. (A, E, and F) Asterisk color indicates comparator as follows: black, N2; green, *daf-2*; and purple, *daf-16*. See also Figure S5.

Reduced insulin/IGF-1 signaling (IIS) increases lifespan in *C. elegans* via proximate mechanisms that remain unclear (Kenyon, 2010) but likely entail slower development of the senescent pathologies that cause death. However, the existence of a definable process of organismal death suggests an additional possibility: that in IIS mutants a given level of senescent pathology is less likely to trigger organismal death, i.e., they are to some degree death resistant. Consistent with this, mutation of the *daf-2* insulin/IGF-1 receptor reduces DF (Coburn et al., 2013), and shrinking during death in senescent worms is suppressed by *daf-2* RNAi (Stroustrup et al., 2013). Exploring this further, we found that *daf-2(e1370)* reduced the magnitude of DC in young adults, after killing with either tBOOH or heat (Figure 6A; Figure S5A).

All *daf-2* mutant alleles can cause increased longevity, but some (such as *e1370*) show multiple pleiotropic effects that can be dissociated from effects on lifespan (Gems et al., 1998). We therefore compared DC resistance in two non-pleiotropic (class 1) alleles, *e1368* (weaker) and *m41* (stronger), and two pleiotropic (class 2) mutants, *e1370* and *m579* (similar severity) (20°C), and we observed DC resistance in all but *e1368* (Figure 6B). The DC resistance of *daf-2(m41)* shows that this is not a class 2-specific pleiotropic trait. That *e1370* increases lifespan more than *e1368* (20°C) (Podshivalova et al., 2017) supports a possible role of DC resistance in *daf-2* longevity.

*daf-2* mutant longevity requires the DAF-16 FoxO transcription factor (Kenyon, 2010). Surprisingly, reduction of DC in the *daf-2* mutant was largely *daf-16* independent (Figure 6A). This, however, does not rule out the contribution of possible death resistance to increased *daf-2* longevity (i.e., *daf-16* may be necessary for *daf-2* death resistance to increase lifespan). Other downstream mediators of *daf-2* effects on lifespan include the SKN-1/Nrf2 transcription factor, which activates expression of the antioxidant enzymes of the biotransformation system (Tullet et al., 2008), and the PQM-1 transcription factor (Tepper et al., 2013). However, neither are required for *daf-2* DC resistance (Figure 6C). Mutation of *daf-2* increases DAF-16, SKN-1, and PQM-1 activity by reducing levels of phosphatidylinositol (3,4,5)-trisphosphate (PIP3), and this effect is counteracted by mutation of the PIP3 phosphatase DAF-18 (PTEN) (Mihaylova et al., 1999). Notably, *daf-2(e1370)* DC resistance was largely abrogated by *daf-18(nr2037)* (Figure 6C), implying that PIP3 promotes organismal death via another effector pathway (Figure 6D).

A further possibility is that *daf-2(e1370)* promotes DC resistance by maintaining ATP levels in stressful conditions. Consistent with this, *daf-2* caused a delay in the decline in muscle ATP during tBOOH-induced death (compare Figure 5B and Figure S5B); however, this delay may also be attributable to slower tBOOH uptake or increased detoxification (see below). It was previously noted that *daf-2(e1370)* increases overall ATP content in *C. elegans* (Houthoofd et al., 2005). However, ATP levels in muscle in young *daf-2* adults were not increased (Figure S5C).

*daf-2(e1370)* also caused a significant delay in the onset of both DC and DF after tBOOH exposure (Figures 6E and 6F; Table S1). This is consistent with the known *daf-2* mutant resistance to tBOOH (Tullet et al., 2008). It is unclear how reduced IIS suppresses DC and DF, but *daf-2* did not detectably reduce the magnitude of the death-associated increase in sarcoplasmic Ca^2+^ (Figure S5D). Whether DC resistance contributes to *daf-2* longevity remains to be demonstrated.

### Evidence that Ca^2+^-Dependent Osmotic Effects Promote Recovery from DC

As in mammalian *rigor mortis*, DC in *C. elegans* is followed by recovery, where the body and muscle sarcomeres regain their initial length (Figure 1A; Movie S1). In mammals, recovery is promoted by muscle proteolysis caused by Ca^2+^-dependent proteases (calpains) (Geesink et al., 2006, Huff Lonergan et al., 2010, Kent et al., 2004), although this has little effect on the migration properties of myosins on SDS-PAGE (Huff Lonergan et al., 2010); similarly, we saw no signs of myosin degradation during recovery in tBOOH-induced death (Figure S6A). To probe whether recovery from DC in *C. elegans* is mediated by calpain-mediated proteolysis, we tested for the effects of overexpression or deletion of *clp-1*, the major muscle calpain in *C. elegans* (Joyce et al., 2012), but none was detected (Figure S6B). Possibly other muscle-expressed proteases promote recovery from DC.

Both *rigor mortis* and recovery from it are promoted by Ca^2+^ and, therefore, chelation of Ca^2+^ is predicted to inhibit both. Ca^2+^ chelation with EGTA inhibits *rigor mortis* in vertebrate muscle (Feinstein, 1966, Weiner and Pearson, 1969). We observed that EGTA (400 mM) increased rather than decreased tBOOH-induced DC, but it suppressed recovery (Figure S6C). By contrast, an iso-osmotic solution of NaCl (660 mM) did not suppress recovery, arguing against a non-specific osmotic effect of EGTA. However, 660 mM NaCl did partially suppress recovery after death from heat stress (Figure S6D). We also tested whether EGTA reduces sarcoplasmic Ca^2+^ levels during DC, but, against expectation, it did not (Figure S6E). This implies that the suppression of recovery by EGTA is not due to reduced Ca^2+^-dependent processes in muscle. Overall, these results suggest that a Ca^2+^-dependent osmotic effect contributes to the recovery from DC (discussed further below).

## Discussion

**Figure 7:**
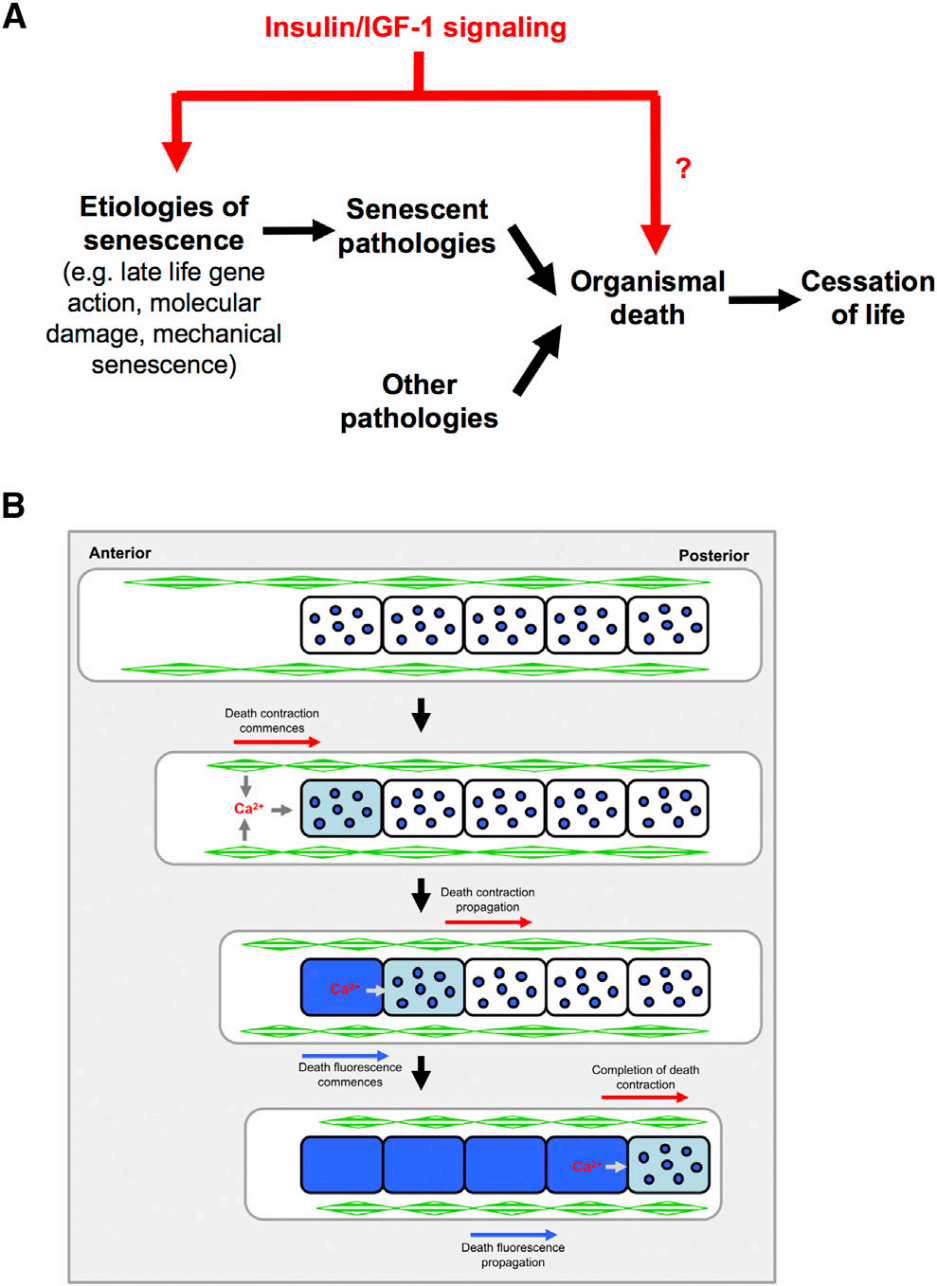
Senescent and Non-senescent Pathology and Organismal Death (A) Model describing the place of organismal death in the chain of events that determine lifespan and describing hypothetical dual action of IIS in lifespan determination. (B) Model describing coupling of DC, Ca^2+^, and DF waves, promoting muscular and intestinal necrosis and organismal death. One possibility is that Ca^2+^ released from dying muscle cells during *rigor mortis* leaks onto the anterior intestine (jumping Ca^2+^ wave model) triggering necrosis and DF. Green diamonds, body wall muscle; blue circles, lysosome-like organelles (gut granules) loaded with blue fluorescent anthranilates. See also Figure S7.

The process of organismal death and how it is triggered by aging are not well understood in mammals and barely at all in *C. elegans.* Three major mysteries relating to death from old age are as follows: how senescence generates pathologies that cause death, how these pathologies trigger death, and the mechanisms of organismal death itself (Figure 7A). This study yields insights into the two latter issues, implicating Ca^2+^ release and ATP depletion as triggers of organismal death and suggesting a process in which coupled waves of *rigor mortis* and fluorescence-marked intestinal necrosis are propagated along the organism, in a terminal process of cellular destruction (Figure 7B; Figure S7).

### A *Rigor Mortis*-like Process Occurs during Organismal Death in *C. elegans*

We previously demonstrated that, in dying *C. elegans*, there is a wave of intestinal necrosis accompanied by DF (Coburn et al., 2013). Findings presented here show that organismal death in *C. elegans* includes events similar to mammalian *rigor mortis*. Both involve muscle hyper-contraction at death, accompanied by reduced ATP and increased Ca^2+^; both are followed by recovery from hyper-contraction; and both show a decline in magnitude with increasing age as the result of sarcopenia. However, there are also some differences. First, in mammals, *rigor mortis* occurs some time after the main events in organismal death (cessation of heart function and brain death), whereas in *C. elegans* it occurs earlier. This may reflect the fact that, in mammals, anoxia resulting from the loss of circulation promotes organismal death, whereas *C. elegans* does not possess a vascular system due to its small size and, therefore, anoxia is not expected to play a role in their death; hence *rigor mortis* is a relatively early event during death (Figure S7). Second, in mammals, recovery from *rigor mortis* is promoted by muscle proteolysis, whereas we were unable to detect a role of proteolysis in recovery from DC in *C. elegans*; instead, our results suggest that recovery from DC is a Ca^2+^-dependent osmotic effect (see below for further discussion).

### DC and Intestinal Necrosis Are Coupled

Findings presented here show that DC is coupled with and slightly precedes DF. This suggests that DC can trigger the intestinal necrosis that generates DF, i.e., that death can spread from the musculature to the intestine. *Rigor mortis* and necrosis are similar processes, involving transmembrane gradient collapse and Ca^2+^ influx into the cytoplasm that promotes proteolytic (e.g., calpain-mediated) destruction (Koohmaraie, 1992, Yamashima and Oikawa, 2009); consistent with this, DC and DF are both accompanied by a Ca^2+^ wave.

In AP DC waves, the muscle Ca^2+^ wave first approaches the intestine at its anterior end. One possibility is that a Ca^2+^ leak from dying muscle cells onto anterior intestinal cells triggers the AP DF wave, i.e., the Ca^2+^ wave jumps from the body wall muscle (or possibly pharyngeal muscle) to the intestine (Figures 4E and 7B). An additional potential trigger is the pharyngeal-intestinal collision caused by head muscle hyper-contraction (Figures 4A and 4B).

The coupling of DC and intestinal necrosis suggests a link between the biology of organismal death and that of defecation. During the latter, peristalsis is affected by a PA intestinal Ca^2+^ wave (Peters et al., 2007) that is coupled to muscle contraction by proton release (Beg et al., 2008). Overall, these results support a model of *C. elegans* organismal death in which a Ca^2+^-triggered wave of terminal muscle contraction triggers a second, coupled wave of intestinal necrosis, in a process that in some respects resembles a distorted and destructive defecation cycle.

### Osmotic Swelling as a Possible Driver of Recovery from DC

The apparent differences between vertebrates and *C. elegans* in the mechanisms of recovery from DC may in part reflect differences in size and anatomy. For example, in vertebrates, muscular elongation during recovery from *rigor mortis* is powered by tension transmitted from muscle hyper-contraction to ligaments and bones. Proteolytic degradation of muscle allows this elongation to occur. Although *C. elegans* has no bones, it does possess a hydrostatic skeleton and exoskeleton (cuticle) against which muscles work (Karbowski et al., 2006). *C. elegans* corpses when handled appear limp and floppy, suggesting that the hydrostatic skeleton collapses during organismal death. Moreover, worm pick-handling observations suggest that worms during the throes of DC do not develop rigidity (data not shown). In the absence of the hydrostatic skeleton, it is not clear what drives the elongation of the worm during recovery.

One possibility is that recovery is driven by the effect of osmotic swelling in the intestine, occurring as the result of intestinal necrosis, or osmotic swelling more generally throughout the worm. That EGTA, but not an iso-osmotic concentration of NaCl, suppresses recovery could imply that Ca^2+^-dependent intestinal necrosis leads to osmotic swelling, which promotes recovery. Whether proteolysis of muscle plays a permissive role in recovery from DC warrants further investigation.

### Defining Senescent Death and Its Origins

It is difficult to define exactly when cells and organisms become inexorably committed to the process of death. One view is that the point of no return is the inability to maintain transmembrane gradients and, particularly, those that control Ca^2+^ levels (Orrenius et al., 2003).

Maintenance of transmembrane gradients requires ATP, but our findings did not support the simple model that gradual age-related decline in metabolic capacity leads to ATP depletion and, consequently, death. Instead, ATP levels are well maintained into late life, beyond the age at which many major senescent pathologies appear (Ezcurra et al., 2017). By contrast, during organismal death caused by tBOOH or senescence (class C animals that include the dying), ATP levels drop dramatically. One possibility is that this reflects the loss of regulation of ATP production or, more likely, of consumption in dying cells. The end-of-life crash in ATP level could be a trigger and/or a consequence of terminal necrosis in muscle and intestine; this warrants further investigation.

Crashes in ATP level during organismal death have also been described in mammals. For example, in one study of death in dogs, ATP levels in the cerebral cortex dropped by ∼70% within 5 min of cessation of movement (Sarkisian and Adamian, 1970). Moreover, it is well documented that ATP decline and subsequent Ca^2+^ overload mediate the necrotic cell death and excitotoxicity-induced cell death that accompanies stroke and Alzheimer’s and Parkinson’s diseases (Gorman, 2008). Thus, studies of pathology, ATP, Ca^2+^ signaling, and organismal death in *C. elegans* may provide insights into these fundamental mechanisms of pathophysiology.

### Conclusions

In this study, we have documented a phenomenon that is part of the process of organismal death in *C. elegans*, accompanying intestinal necrosis (and death fluorescence): DC, which is driven by muscle hyper-contraction and shares features with mammalian *rigor mortis*. DC is accompanied by a decline in ATP levels and increased Ca^2+^, and it is coupled to intestinal necrosis, which it often precedes and, potentially, triggers, in what resembles a distorted and destructive defecation cycle. These findings show how *C. elegans* can be used as a model to understand conserved mechanisms of organismal death, and they raise questions such as what are the pathologies that trigger organismal death and how do they do so and is resistance/susceptibility to organismal death a determinant of *C. elegans* lifespan?

## Experimental Procedures

### Worm Maintenance and Strains

All strains were maintained at 20°C on nematode growth medium (NGM) plates seeded with *E. coli* OP50 as a food source (Brenner, 1974), unless otherwise specified. For wild-type, hermaphrodites of the N2 male stock (N2 CGCM) were used (Gems and Riddle, 2000). Other strains included the following: AQ2953 *ljIs131[pmyo-3::GCaMP3-SL2-TagRFP-T]*, CB66 *unc-22(e66) IV*, CB190 *unc-54(e190) I*, CB723 *unc-60(e723) V*, CD1035 *daf-2(e1370) III; skn-1(zu135) IV*, CQ200 *pqm-1(ok485) II; daf-2(e1370)) III*, DR1563 *daf-2(e1370) III*, DR1564 *daf-2(m41) III*, DR1566 *daf-2(m579) III*, DR1572 *daf-2(e1368ts) III*, EU31 *skn-1(zu135) IV*, GA47 *unc-15(e73) I*, GA111 *daf-2(e1370) III; daf-18(nr2037) IV*, GA2000 *clp-1(tm690) III*, GA2001 *wuIs305[pmyo-3::Queen-2m]*, GA2002 *daf-2(e1370) III; wuIs305[pmyo-3::Queen-2m]*, GA2003 *daf-16(mgDf50) I; daf-2(e1370) III; wuIs305[pmyo-3::Queen-2m]*, GR1307 *daf-16(mgDf50) I*, HT1890 *daf-16(mgDf50) I; daf-2(e1370) III*, NS3227 *daf-18(nr2037) IV*, PK2724 *crIs4 punc-54::clp-1::myc psur-5::gfp,* RB711 *pqm-1(ok485) II,* RW1596 *myo-3(st386) V; stEx30[pmyo-3::GFP + rol-6(su1006)]*, and VK689 *vkIs689 [pnhx-2::sGFP::ATM + pmyo-2::mCherry]*.

### DC Assays

For tBOOH-induced death, animals were placed in a drop of 14% tBOOH and observed using time-lapse photography. For heat-induced death, animals were placed onto a thermoelectrically heated microscope stage (PE120, Linkam Scientific) and heated to 52.5°C. Images were captured every 30 s for 2 hr for tBOOH-induced death and every 2.5 s for 2 min for heat-induced death. Changes in worm body length were analyzed by converting multiple images into kymographs, which were then analyzed using a MATLAB script (MathWorks) (Data S1; for additional details, see the Supplemental Experimental Procedures).

Time-lapse photography was also performed on animals dying of old age. Barely mobile N2 animals at an advanced stage of senescence (late class C) (Coburn et al., 2013) were picked, and images were taken every 15 min for 4–20 hr as the worms expired. We also assessed DC, DF, and Ca^2+^ increases during death from old age in the strain AQ2953 expressing GCaMP3 in body wall muscles. AQ2953 worms were grown at 25°C with *pos-1* RNAi in a custom automated vermiculture system (Zhang et al., 2016b), and images were captured every 15 min over a 6-day period in late life, during which the majority of animals died of old age. Assessment of presence and orientation of DC, Ca^2+^, and DF waves was performed manually and independently by two people; for additional details, see the Supplemental Experimental Procedures.

### Calcium Imaging

For Ca^2+^ imaging, the strain AQ2953 was used, which expresses GCaMP3 together with TagRFP in body wall muscles. DF was observed using a DAPI filter cube, GCaMP3 with a GFP filter cube, and TagRFP with a Rhodamine filter cube. In each worm, the GCaMP3/TagRFP ratio for a randomly selected region of head muscle was measured prior to death and at its subsequent maximal level. GCaMP3/TagRFP induction was then calculated as a ratio of GCaMP3/TagRFP_Max_ and GCaMP3/TagRFP_Initial_.

### ATP Measurements

To assay age changes in ATP content per worm, a CellTiter-Glo Luminescent Cell Viability Assay (Promega) was used with N2 worms of a range of ages (5 worms per assay) and in worms of different motility classes (A, B, and C) (1 worm per assay, n = 10 per trial). For estimates of relative ATP levels using the Queen-2m sensor, λ_ex_ of 402/15 nm or 482/28 nm and λ_em_ of 545/25 was measured and 402/482 excitation ratio calculated, as described (Yaginuma et al., 2014); for additional details, see the Supplemental Experimental Procedures.

### Statistical Analysis

Data mean values are presented ± SEM. For tests of statistical significance, a one-way or two-way ANOVA with Tukey’s honest significant difference (HSD) correction or a two-tailed t test was performed.

## Supplemental Information

Document S1. Supplemental Experimental Procedures and Figures S1–S7

Table S1. Summary Statistics for Measurements of Death-Related Phenomena, Related to Figures 1, 2, 3, 4, 5, 6, S3, S5, and S6

Movie S1. Anterior-to-Posterior Wave of Contraction and Death Fluorescence (a Marker of Necrosis) during tBOOH-Induced Death in Young Adult Hermaphrodite Expressing *pmyo-3::GFP* to Show Body Wall Muscles, Related to Figure 1 Real time: 34 min

Movie S2. Wave of Contraction and Relaxation in N2 Hermaphrodite Dying as the Result of Senescence, Related to Figure 1 Real time: 15 hr

Movie S3. Death Contraction in the Head Drives the Pharynx Backward into the Anterior Intestine (Belly Punch Effect) in a Young Adult Hermaphrodite, Apparently Triggering Death Fluorescence (a Marker of Necrosis) in the Anterior Intestine, Related to Figure 4 Real time: 34 min

Movie S4. tBOOH-Induced Death Causes an AP Wave of GCaMP3 Ca^2+^ Fluorescence in Muscle in a Young Adult Hermaphrodite, followed Immediately by a Wave of Death Fluorescence (a Marker of Necrosis), Related to Figure 4 Real time: 19 min

Movie S5. ATP Decline Measured by ATP Sensor Queen-2 m Expressed in Muscles during tBOOH-Induced Death in a Young Adult Hermaphrodite, Related to Figure 5 Real time: 17 min

Data S1. MATLAB Script to Extract Coordinates Corresponding to the Head and the Tail Ends from Kymographs and R Scripts to Analyze Body Length Changes, Related to Death Contraction Assays in the Experimental Procedures

Document S2. Article plus Supplemental Information
