# Supplementary material for: Coupling of Rigor Mortis and Intestinal Necrosis during C. elegans Organismal Death
Source: Cell Rep. 2018 Mar 6;22(10):2730–41. doi: 10.1016/j.celrep.2018.02.050 (PMC5863043; doi:10.1016/j.celrep.2018.02.050)
Supplement: Document S1. Supplemental Experimental Procedures and Figures S1–S7 [file mmc1.pdf]

Cell Reports, Volume *22*

Supplemental Information

Coupling of *Rigor Mortis* and Intestinal Necrosis during *C. elegans* Organismal Death

Evgeniy R. Galimov, Rosina E. Pryor, Sarah E. Poole, Alexandre Benedetto, Zachary Pincus, and David Gems

SUPPLEMENTAL INFORMATION

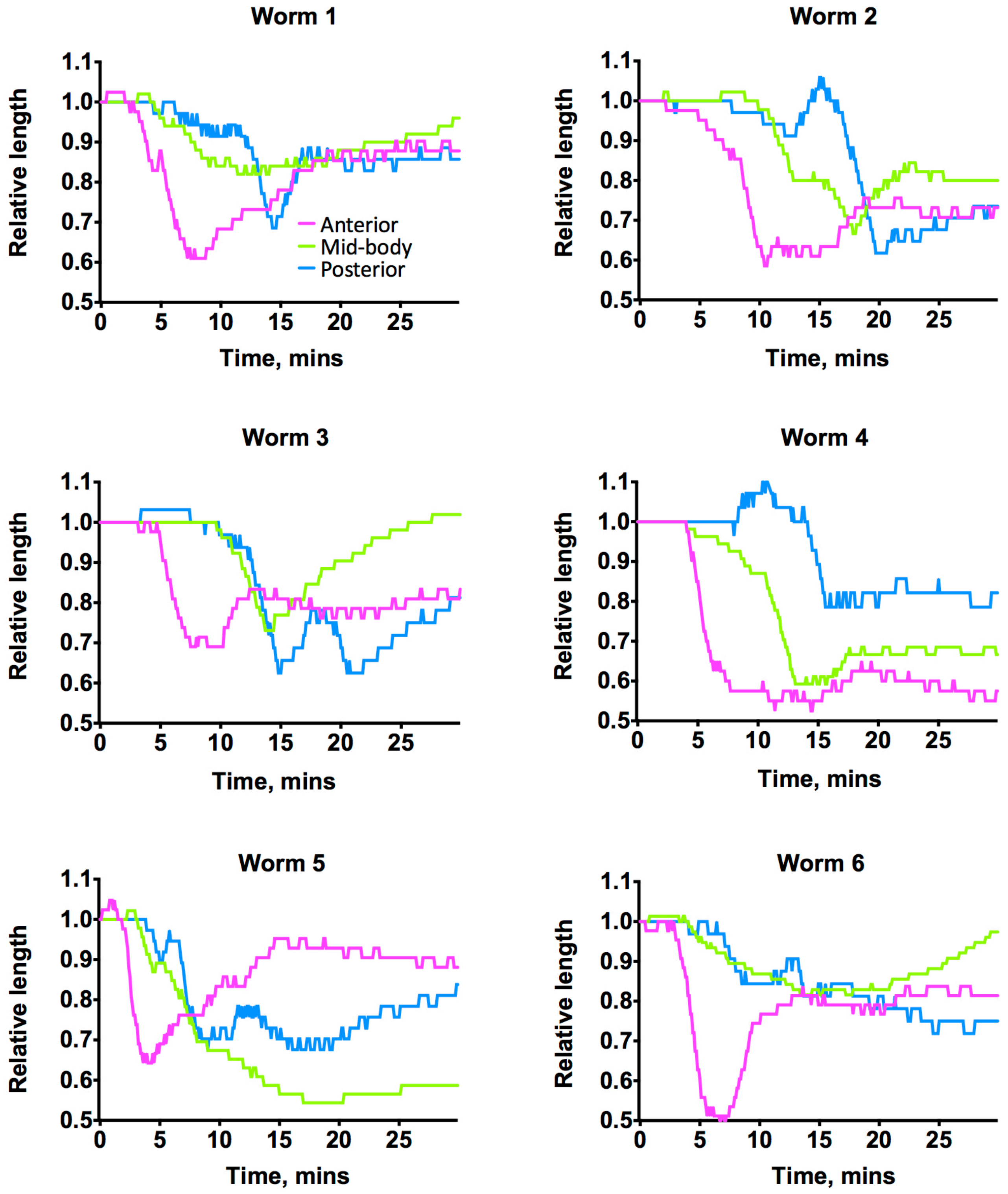

Figure S1. Longitudinal Body Contraction at Death. Related to Figure 1.
Data from six randomly selected individual young adults killed with tBOOH, showing contraction in anterior, mid-body and posterior regions. Note the high degree of inter-individual variability in the contraction; however, the anterior to posterior progression of the contraction is distinguishable in all cases.

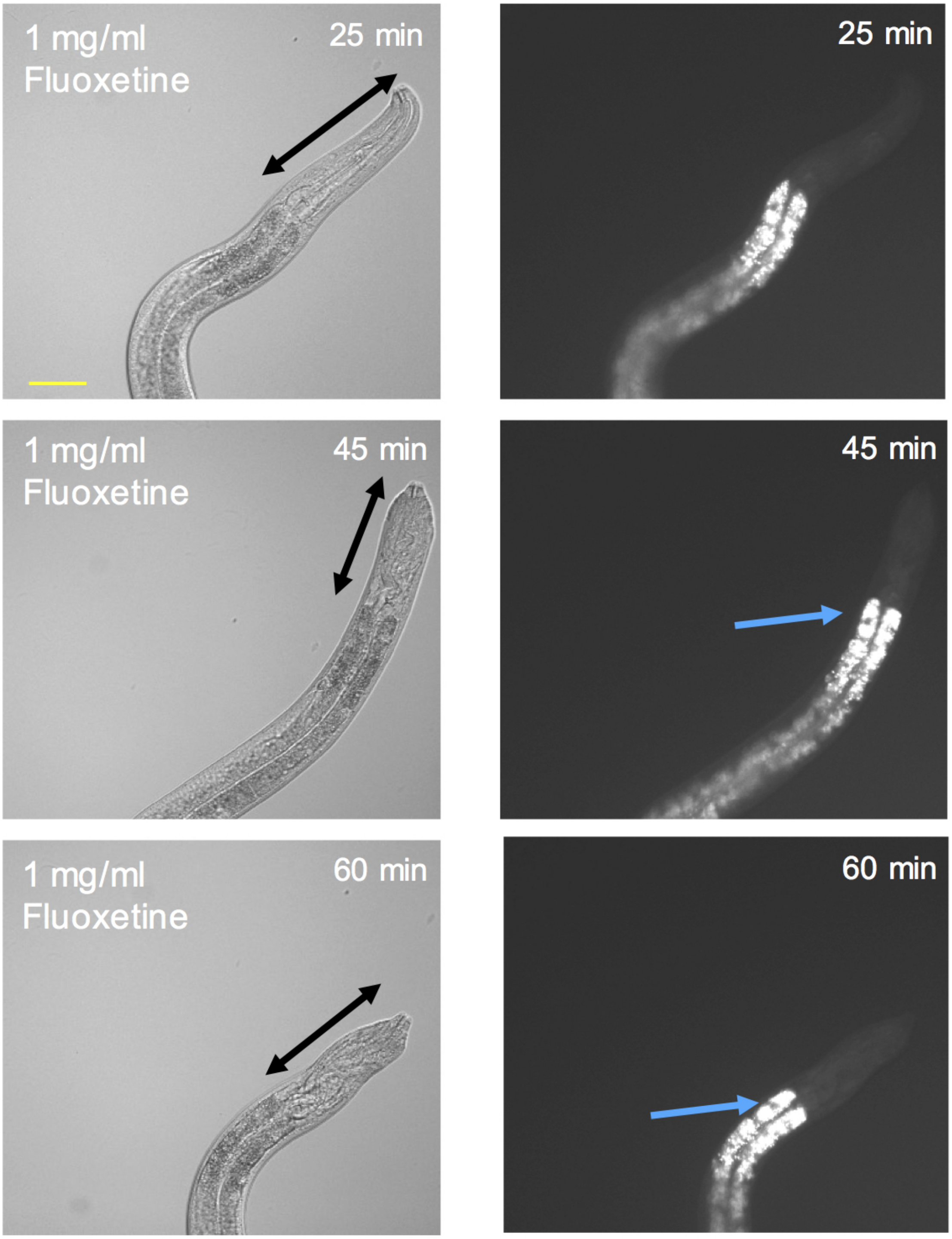

Figure S2. Fluoxetine Induces Nose Contraction but not Death Fluorescence. Related to Figure 1.
This distinguishes fluoxetine-induced nose muscle contraction (black arrows) from death contraction, i.e. death contraction in response to tBOOH is not a chemosensory response of this type. The absence of death fluorescence is indicated by the lack of change in punctate anthranilate autofluorescence in the intestine (blue arrows).

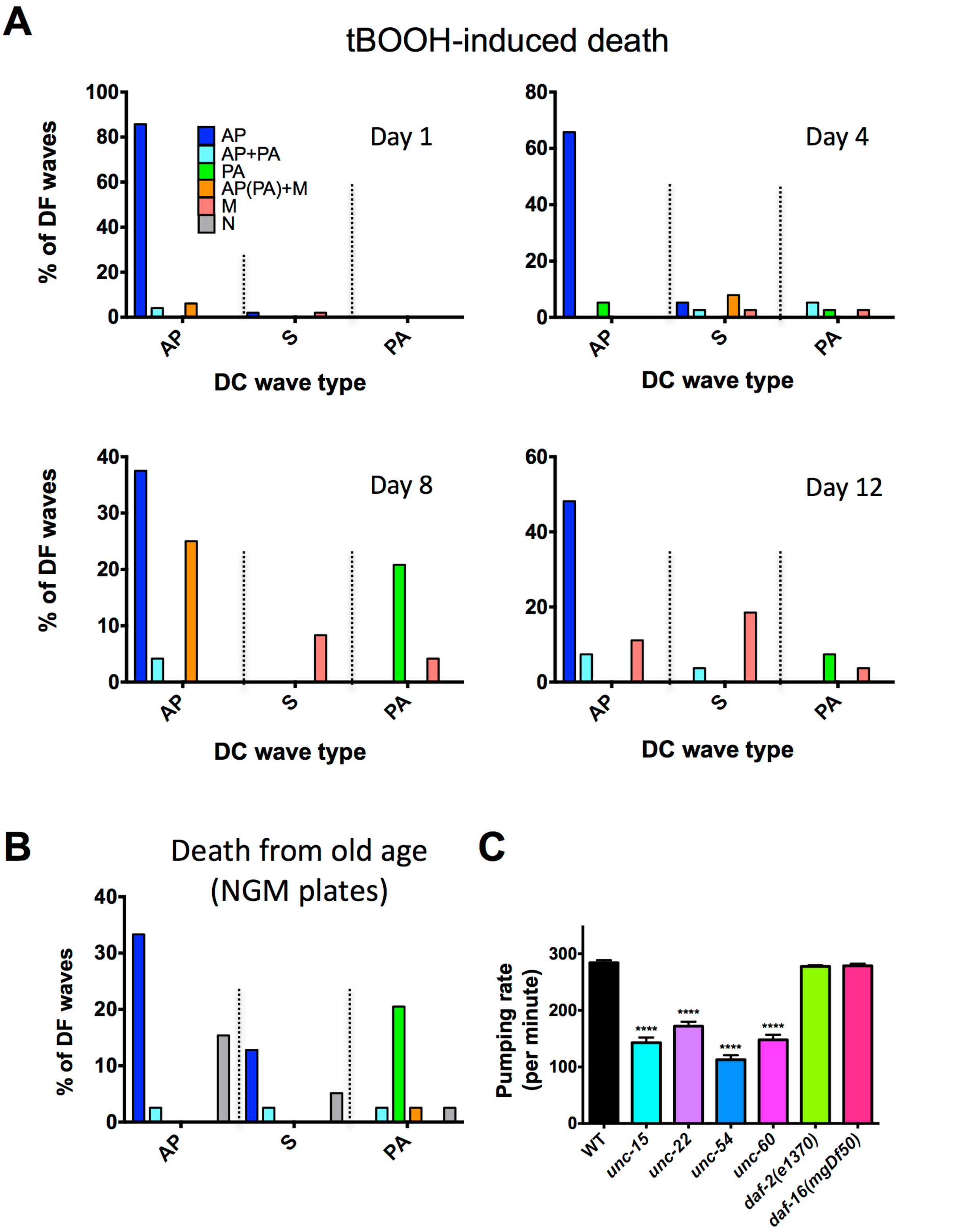

Figure S3. Correlations Between DC and DF Waves. Related to Figure 3.
(A) tBOOH-induced death. (B) Senescence-induced death. n = 13-41, pooled from 3-8 experiments. AP, anterior-to-posterior waves; S, simultaneous anterior and posterior contraction; PA posterior-to-anterior waves; M, waves originating in the mid-body and propagating outwards; N, no contraction or no increase in fluorescence. (C) Pharyngeal pumping rate in selected mutants. n = 40-100, pooled from 4-6 experiments. Error bars, S.E.M.. ****$p < 0.0001$; one-way ANOVA.

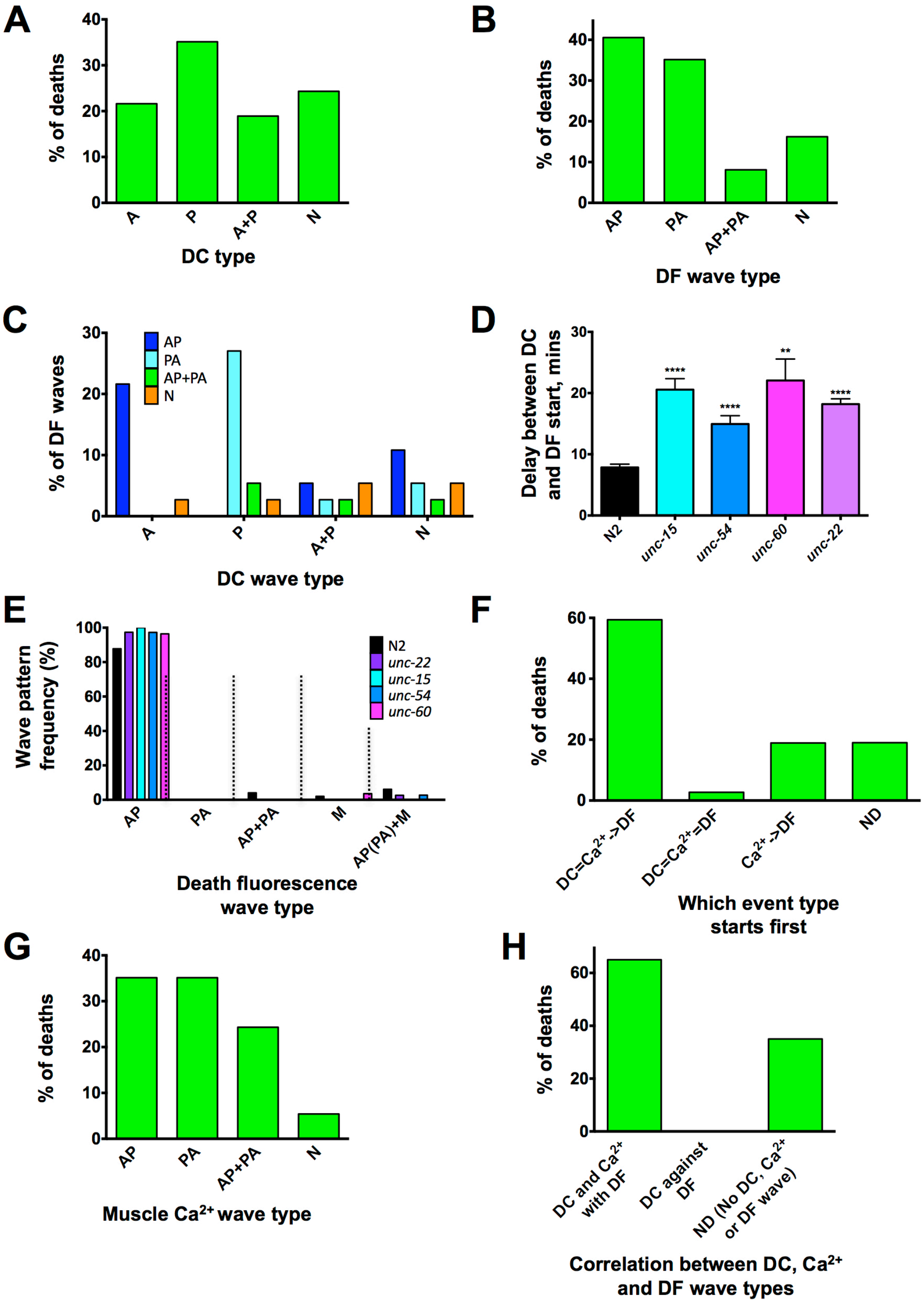

Figure S4. Correlations between DC, DF and Ca^{2+} Waves. Related to Figure 3 and Figure 4.
(A-C, F-H) Data from worms dying of old age (automated vermiculture system); AQ2953 grown at 25˚C and rendered sterile by *pos-1* RNAi. (A) DC. (B) DF. A, anterior; P, posterior; A+P, anterior and posterior, N, no clear wave. (C) Correlations between DC and DF waves. (D) Delay between DC and DF start time in N2 and muscle-deficient strains. (E) DF waves distribution among muscle-deficient strains. (D,E) D1 adults killed with tBOOH (n = 14-29 pooled from 4-6 experiments; ** $p < 0.01$; **** $p < 0.0001$; one-way ANOVA). (F) Determining the order of events during death: $DC=Ca^{2+}$->DF: DC and Ca^{2+} increase happen simultaneously then DF; $DC=Ca^{2+}$=DF: DC and Ca^{2+} appear simultaneously with DF; Ca^{2+}->DF: Ca^{2+} increase happen first then DF (no DC detected); ND: Ca^{2+} or DF is absent. (G) Frequency of AP, PA, or AP+PA, waves of Ca^{2+} increase in worms dying of old age. (H) Correlation between direction of DC, Ca^{2+} and DF. A-C, F-H: n = 37.

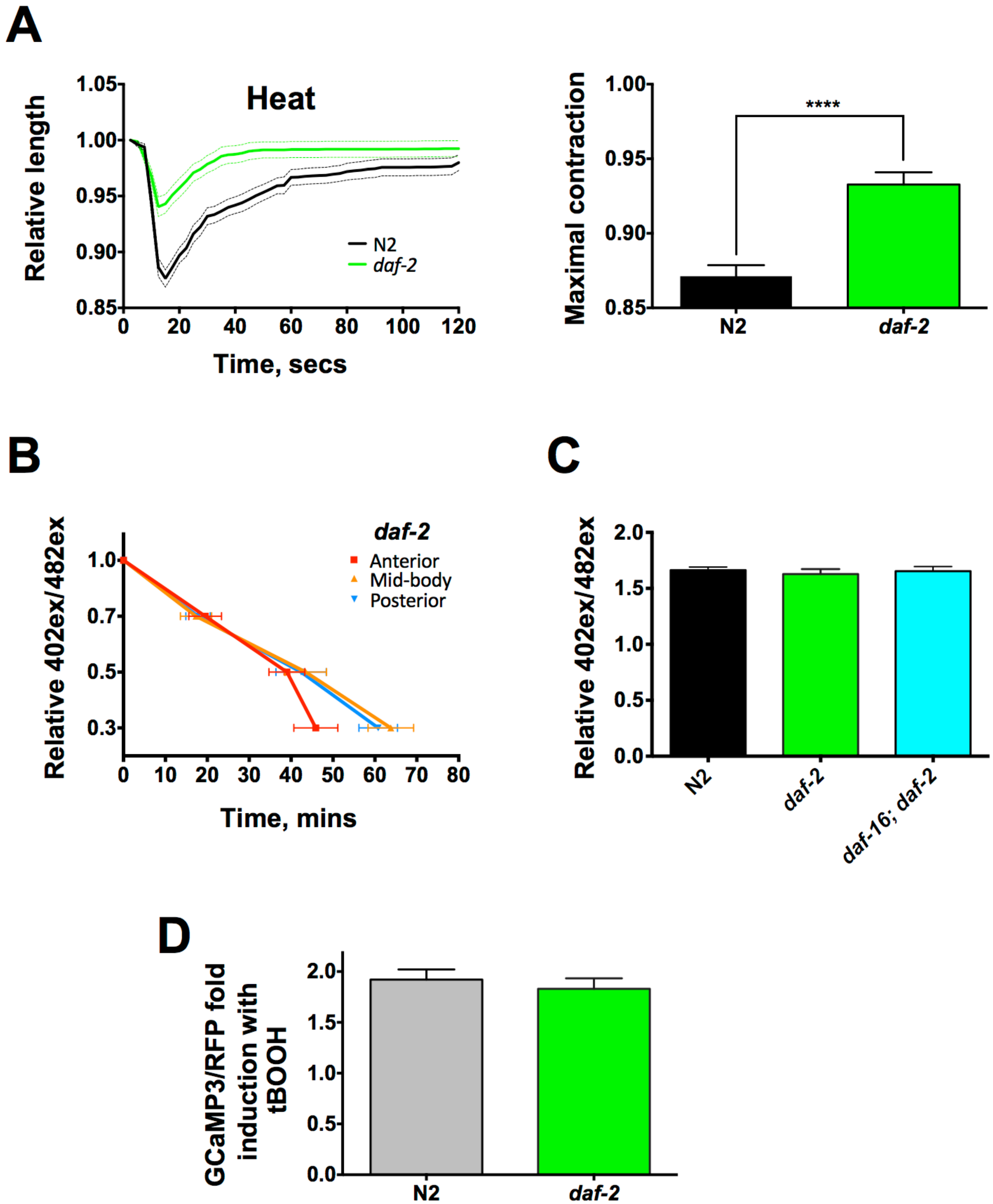

Figure S5. **Effects of *daf-2* on the Biology of Death Contraction. Related to Figure 5 and Figure 6.**
(A) Death from heat stress in *daf-2* mutants. Left, DC curves; right, maximal DC (*n* = 13-18; pooled from 5-6 experiments; **** $p < 0.0001$; one-way ANOVA). (B) ATP decline in muscles in *daf-2* mutants (d1 adults) measured with Queen-2m upon tBOOH killing in different parts of nematode body (*n* = 10; pooled from 2 experiments, two-way ANOVA with Tukey's HSD correction). (C) Muscle ATP level in N2, *daf-2* and *daf-16; daf-2* mutants (d1 adults) measured with Queen-2m (*n* = 48-51, pooled from 2 experiments). A-C, one-way ANOVA. (D) Ca^{2+} levels (GCaMP3 fluorescence) in anterior body wall muscle cytoplasm during tBOOH-induced death in N2 and *daf-2(e1370)* (n = 25-27, pooled from 6 experiments; $p > 0.05$, unpaired t test).

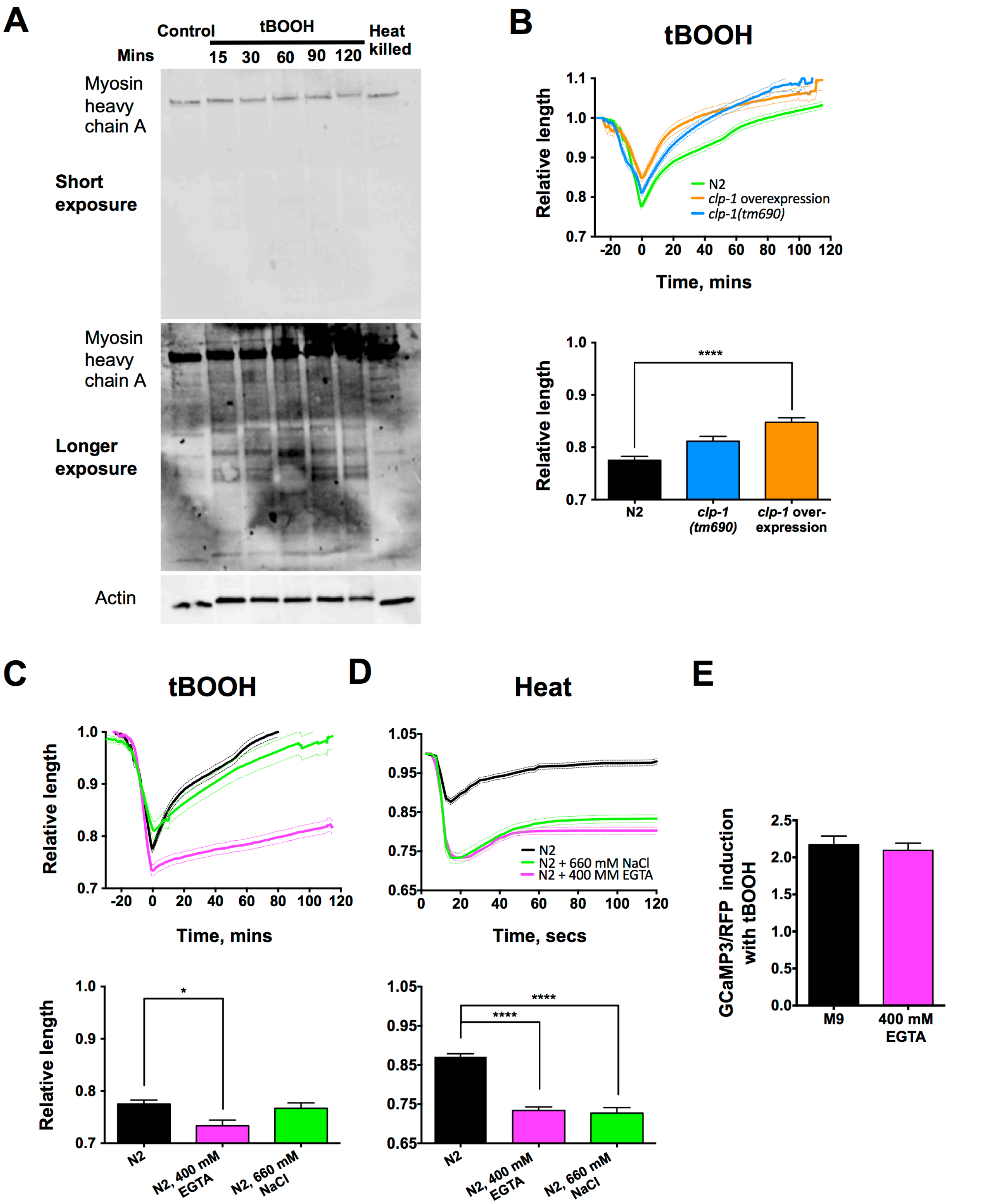

Figure S6. **Determinants of Recovery after DC. Related to Figure 1 and Figure 4.**
(A) No detectable breakdown of muscle myosin heavy chain A during tBOOH-induced death. Western blots of whole young adult hermaphrodite extracts (10 animals/sample) stained with antibodies against myosin heavy chain A and actin; right: same Western blots, longer exposure for anti-myosin heavy chain A (N = 2). (B) Effect of *clp-1(tm690)* (loss of function) and *crIs4 punc-54::clp-1* (over-expression) on tBOOH-induced DC and recovery. (C,D) Ca^{2+} chelation with 400 mM EGTA enhances DC but suppresses recovery during death. (C) tBOOH-induced death. (D) Heat-induced death. 660 mM NaCl, iso-osmotic control. (E) No detectable reduction of muscle Ca^{2+} by EGTA; GCaMP3/RFP ratio in tBOOH-killed worms (n = 15, pooled from 2 experiments; unpaired t test). B-D, n = 13-22, pooled from 3-6 experiments; one-way ANOVA. Error bars, S.E.M.. *$p < 0.05$; ****$p < 0.0001$.

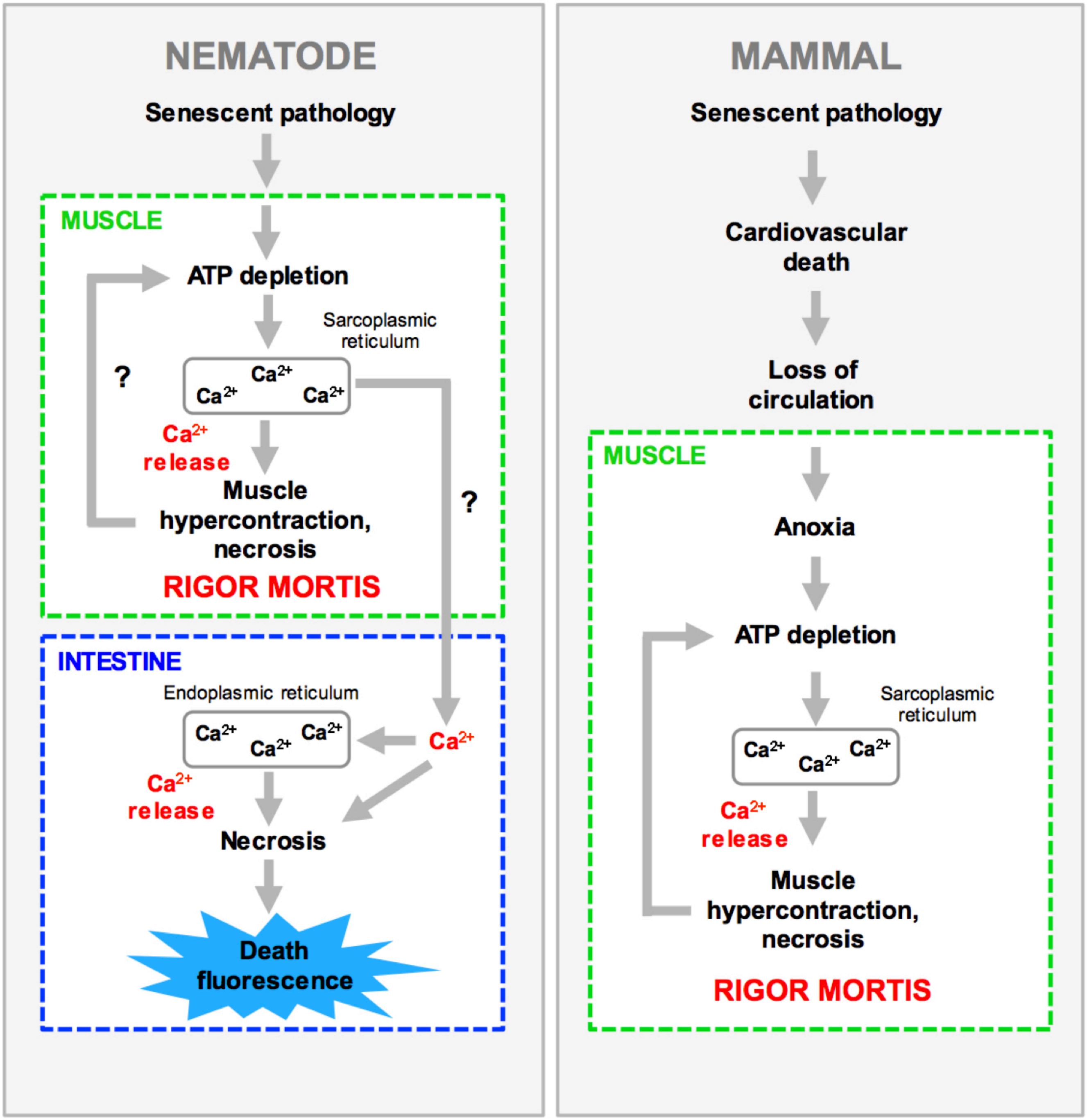

Figure S7. Hypothetical Model of Organismal Death in *C. elegans* and its Relation to Mammalian Rigor Mortis. Related to Figure 7.
In mammals rigor mortis occurs after cessation of heart function leading to anoxia and ATP depletion, which in turn causes Ca^{2+} increase in cytoplasm and chronic muscle contraction. Rigor mortis is an earlier event in organismal death in *C. elegans*, which lack a vascular system. One possibility is that rigor mortis in *C. elegans* is also triggered by ATP depletion resulting from as yet unidentified pathology; an additional possibility is that ATP consumption in hypercontracted muscle further depletes ATP, as has been proposed to occur in mammalian rigor mortis. We speculate that the resulting increase in sarcoplasmic Ca^{2+} its leakage from dying muscle cells onto the anterior intestine triggers intestinal necrosis.

SUPPLEMENTAL EXPERIMENTAL PROCEDURES

Microscopy

Nomarski and epifluorescence microscopy were performed using either a Leica DM RXA2 microscope (filter cubes: DAPI, λ_{ex} 360/40 nm, λ_{em} 470/40 nm; GFP, λ_{ex} 470/40 nm, λ_{em} 525/50 nm; rhodamine, λ_{ex} 546/12 nm, λ_{em} 600/40 nm) connected to a Hamamatsu C10600 - Orca ER digital camera; or a Zeiss Axioskop2plus microscope (filters: DAPI, λ_{ex} 402/15 nm, λ_{em} 455/50 nm; GFP, λ_{ex} 482/28 nm, λ_{em} 525/36 nm; rhodamine, λ_{ex} 545/25 nm, λ_{em} 605/70 nm) connected to a Hamamatsu C4742-95 digital camera. For image acquisition (including time lapse) and quantification the application Volocity 5.2 (Improvision, Perkin Elmer) was used.

Fluoxetine Nose Contraction Assay

1 day old adults were incubated with 1 mg/ml fluoxetine (Sigma-Aldrich, F132) on 2% agarose pad for the indicated period of time. Bright field and blue epifluorescence (DAPI cube) images were captured using a Leica DM RXA2 microscope system.

Death Contraction Assays

tBOOH-induced death: animals were placed in a drop of 14% tBOOH in M9 buffer on a 2% agarose pad on a microscope slide, and observed using time-lapse photography. To study the effects of EGTA on DC a 400 mM EGTA solution containing 14% tBOOH was used. As a control, 14% tBOOH and 660 mM NaCl solution was used (660 mM NaCl generates the same osmotic pressure as 400 mM EGTA). Heat-induced death: animals were placed in a drop of M9 on a 2% agarose pad on a microscope slide, which was then placed onto a thermoelectrically-heated microscope stage (PE120, Linkam Scientific), and heated to 52.5˚C. Images were captured every 30 secs for 2 hr for tBOOH-induced death, every 2.5 sec for 2 min for heat-induced death. Images were captured at 2.5x magnification, and DF was observed using a DAPI filter cube (Coburn et al., 2013).

Changes in worm body length were analyzed by converting multiple images into kymographs. A single line was drawn along each worm body along its central axis, and kymographs were created using Volocity software such that 10 pixels = 1 min. The images obtained were extracted in TIFF format. Next color 1 pixel lines corresponding to head, tail and DF wave positions were drawn using Image J (National Institutes of Health, Bethesda, MD, USA). The color code for a line following the head position was (255,0,0), that for a line following the tail position was (0,255,0), and that for a line corresponding to DF was (250,0,0). For AP wave analyses, the same approach was applied; here with two lines corresponding to the head, mid-body and tail regions. Line coordinates were extracted from images using Matlab script (MathWorks, Inc.) and were then used for worm length measurement. Worm length changes as well as times of maximal contraction and start point of DF wave were analyzed using using the software R (2013).

Time-lapse photography was also performed on animals dying of old age. Barely mobile N2 animals at an advanced stage of senescence (late class C) (Coburn et al., 2013) were transferred to a glass bottomed 35 mm µ-Dish (Ibidi) containing a thin layer of NGM agar with a small lawn of *E. coli* OP50, for their comfort (these animals are too old to feed). Bright field and epifluorescence (DAPI cube) images were taken every 15 min for 4-20 hr as the worms expired.

We also assessed DC, DF and Ca^{2+} increases during death from old age in the strain AQ2953 expressing GCaMP3 in body wall muscles. AQ2953 was grown at 25˚C on HT115(DE3) bacteria expressing *pos-1* RNAi in a custom automated vermiculture system (Zhang et al., 2016b), images were taken every 15 mins over a 6 day period in late life during which the majority of worms died of old age. Assessment of presence and orientation of DC, Ca^{2+} and DF waves was performed manually and independently by two people.

Experimental design: For most of the presented findings, data on DC and/or other death-related phenomena was gathered from a series of experiments on small groups (typically 5-7) of dying animals of a given genotype/treatment. The time required of each individual experiments (~2 hrs) meant that in most cases it was not possible to perform trials with simultaneous measurements with sufficiently high sample size for all of the genotypes/treatments being compared. Instead, a series of individual experiments were conducted, and the resulting data pooled to obtain sufficiently large sample sizes. For a full summary of the number of individual experiments and trials performed, and data from each, see Supplemental Table 1.

Belly Punch Analysis

To characterize the relationship between movement of the posterior pharynx into the intestine during DC and the first appearance of DF, the strain VK689 *vkIs689 [nhx-2p::sGFP::ATM + myo-2p::mCherry]* was used. For analysis we employed an invagination score (IS) to calculate the extent of movement into the intestine: IS = I/2R, where I is extent (length) of invagination and R is pharyngeal radius, both measured in pixels.

Construction of ATP Reporter Strain

The Queen-2m sequence was excised from plasmid pRSETB-Queen-2m (kindly provided by H. Imamura) as a *Bam*HI/*Eco*RI restriction fragment and ligated into vector L3785. Transgenic *C. elegans* were then created using microinjection and identified by Queen-2m fluorescence. Extrachromosomal transgene arrays were integrated using γ-irradiation and the resulting lines were subsequently out-crossed at least 6 times.

ATP Measurements

To assay age changes in ATP content per worm, N2 worms of a range of ages (5 worms per measurement, class A worms only examined) were washed and frozen in M9 at -70°C, then lysed at 95°C for 15 mins followed by incubation at 25°C for 5 mins. An equal volume of CellTiter-Glo Luminescent Cell Viability Assay (Promega) reagent was added and mixed. Luminescence was measured over 10 seconds using a Tecan Infinite M200 PRO plate reader. This assay was also used to compare ATP levels in worms of different motility classes (A, B and C), but assays were performed on 10-30 individual worms per trial.

For estimates of relative ATP levels using the Queen-2m sensor, λ_{ex} of 402/15 nm or 482/28 nm and λ_{em} of 545/25 was measured and 402ex/482ex ratio calculated, as described (Yaginuma et al., 2014). To estimate ATP levels in muscles during starvation, worms before or after 36 hr of starvation were immobilized using 0.1 µm diameter polystyrene microspheres (Polysciences 00876-15; 2.5–5% w/v suspension) on 2% agar pads on glass. For other trials Queen-2m epifluorescence in 1 day old adults was measured before and after indicated periods of incubation with 14% tBOOH (Sigma-Aldrich, 458139), 5% 1-phenoxy-2-propanol (Sigma-Aldrich, 484423), 5% sodium azide (Sigma-Aldrich, 71290) or 2 mM oligomycin A (Sigma-Aldrich, 75351). All drugs were dissolved in M9.

Antibodies and Western Blotting

10 x 1-day old wild-type adult hermaphrodites were placed into 14% tBOOH for 15, 30, 60, 90 or 120 min, or heat killed. Then 2x Laemmli loading buffer (S3401, Sigma-Aldrich) containing 5% β-mercaptoethanol was added. The solutions were put at 70˚C for 15 min and then 95˚C for 5 minutes. Proteins were separated on Criterion TGX Stain Free Gel, 4-15% (Bio-rad) and transferred to a nitrocellulose membrane (Amersham). The membranes were then blocked in 5% skimmed milk (Fluka) in PBST (PBS with 0.1% Tween 20) for 1 hr at room temperature, after which they were probed with primary antibodies diluted in 5% skimmed milk (Fluka) in PBST overnight at 4˚C. Blots were developed using the ECL detection system (Amersham). The following primary antibodies were used: mouse anti-actin (Abcam, #ab14128; 1:5000), mouse anti-myosin heavy chain A (DSHB Hybridoma bank, 1:1000).

SUPPLEMENTAL REFERENCES

Feinstein, M.B. (1966). Inhibition of muscle rigor by EDTA and EGTA. Life Sciences *5*, 2177–2186.
Geesink, G., Kuchay, S., Chishti, A., and Koohmaraie, M. (2006). μ-Calpain is essential for postmortem proteolysis of muscle proteins. J. Animal Sci. *84*, 2834-2840.
Huff Lonergan, E., Zhang, W., and Lonergan, S.M. (2010). Biochemistry of postmortem muscle - lessons on mechanisms of meat tenderization. Meat Sci. *86*, 184-195.
Joyce, P.I., Satija, R., Chen, M., and Kuwabara, P.E. (2012). The atypical calpains: evolutionary analyses and roles in Caenorhabditis elegans cellular degeneration. PLoS Genet. *8*, e1002602.
Karbowski, J., Cronin, C., Seah, A., Mendel, J., Cleary, D., and Sternberg, P. (2006). Conservation rules, their breakdown, and optimality in Caenorhabditis sinusoidal locomotion. J. Theor. Biol. *242* 652-669.
Kent, M., Spencer, M., and M. Koohmaraie, M. (2004). Postmortem proteolysis is reduced in transgenic mice overexpressing calpastatin. J. Animal Sci. *82*, 794-801.
Weiner, P., and Pearson, A. (1969). Calcium chelators influence some physical and chemical properties of rabbit and pig muscle. J. Food Sci. *34*, 592-596.
